# Intrauterine Growth and Offspring Neurodevelopmental Traits: A Mendelian Randomization Analysis of the Norwegian Mother, Father and Child Cohort Study (MoBa)

**DOI:** 10.1001/jamapsychiatry.2023.3872

**Published:** 2023-10-25

**Authors:** Shannon D’Urso, Gunn-Helen Moen, Liang-Dar Hwang, Laurie J. Hannigan, Elizabeth C. Corfield, Helga Ask, Stefan Johannson, Pål Rasmus Njølstad, Robin N. Beaumont, Rachel M. Freathy, David M. Evans, Alexandra Havdahl

**Affiliations:** 1 Institute for Molecular Bioscience, The University of Queensland, Brisbane, Queensland, Australia; 2 Institute of Clinical Medicine, Faculty of Medicine, University of Oslo, Oslo, Norway; 3 K. G. Jebsen Center for Genetic Epidemiology, Department of Public Health and Nursing, NTNU, Norwegian University of Science and Technology, Trondheim, Norway; 4 Population Health Science, Bristol Medical School, University of Bristol, Bristol, United Kingdom; 5 Frazer Institute, The University of Queensland, Woolloongabba, Queensland, Australia; 6 Nic Waals Institute, Lovisenberg Diaconal Hospital, Oslo, Norway; 7 Center for Genetic Epidemiology and Mental Health, Norwegian Institute of Public Health, Oslo, Norway; 8 PROMENTA Research Center, Department of Psychology, University of Oslo, Oslo, Norway; 9 Department of Clinical Science, University of Bergen, Bergen, Norway; 10 Department of Medical Genetics, Haukeland University Hospital, Bergen, Norway; 11 Mohn Center for Diabetes Precision Medicine, Department of Clinical Science, University of Bergen, Bergen, Norway; 12 Section for Endocrinology and Metabolism, Children and Youth Clinic, Haukeland University Hospital, Bergen, Norway; 13 Department of Clinical and Biomedical Sciences, Faculty of Health and Life Sciences, University of Exeter, Exeter, Devon, United Kingdom; 14 MRC (Medical Research Council) Integrative Epidemiology Unit, University of Bristol, Bristol, United Kingdom

## Abstract

**Importance:**

Conventional epidemiological analyses have suggested that lower birth weight is associated with later neurodevelopmental difficulties; however, it is unclear whether this association is causal.

**Objective:**

To investigate the relationship between intrauterine growth and offspring neurodevelopmental difficulties.

**Design, Setting, and Participants:**

MoBa is a population-based pregnancy cohort that recruited pregnant women from June 1999 to December 2008 included approximately 114 500 children, 95 200 mothers, and 75 200 fathers. Observational associations between birth weight and neurodevelopmental difficulties were assessed with a conventional epidemiological approach. Mendelian randomization analyses were performed to investigate the potential causal association between maternal allele scores for birth weight and offspring neurodevelopmental difficulties conditional on offspring allele scores.

**Exposures:**

Birth weight and maternal allele scores for birth weight (derived from genetic variants robustly associated with birth weight) were the exposures in the observational and mendelian randomization analyses, respectively.

**Main Outcomes and Measures:**

Clinically relevant maternal ratings of offspring neurodevelopmental difficulties at 6 months, 18 months, 3 years, 5 years, and 8 years of age assessing language and motor difficulties, inattention and hyperactivity-impulsivity, social communication difficulties, and repetitive behaviors.

**Results:**

The conventional epidemiological sample included up to 46 970 offspring, whereas the mendelian randomization sample included up to 44 134 offspring (median offspring birth year, 2005 [range, 1999-2009]; mean [SD] maternal age at birth, 30.1 [4.5] years; mean [SD] paternal age at birth, 32.5 [5.1] years). The conventional epidemiological analyses found evidence that birth weight was negatively associated with several domains at multiple offspring ages (outcome of autism-related trait scores: Social Communication Questionnaire [SCQ]–full at 3 years, β = −0.046 [95% CI, −0.057 to −0.034]; SCQ–Restricted and Repetitive Behaviors subscale at 3 years, β = −0.049 [95% CI, −0.060 to −0.038]; attention-deficit/hyperactivity disorder [ADHD] trait scores: Child Behavior Checklist [CBCL]–ADHD subscale at 18 months, β = −0.035 [95% CI, −0.045 to −0.024]; CBCL-ADHD at 3 years, β = −0.032 [95% CI, −0.043 to −0.021]; CBCL-ADHD at 5 years, β = −0.050 [95% CI, −0.064 to −0.037]; Rating Scale for Disruptive Behavior Disorders [RS-DBD]–ADHD at 8 years, β = −0.036 [95% CI, −0.049 to −0.023]; RS-DBD–Inattention at 8 years, β = −0.037 [95% CI, −0.050 to −0.024]; RS-DBD–Hyperactive-Impulsive Behavior at 8 years, β = −0.027 [95% CI, −0.040 to −0.014]; Conners Parent Rating Scale–Revised [Short Form] at 5 years, β = −0.041 [95% CI, −0.054 to −0.028]; motor scores: Ages and Stages Questionnaire–Motor Difficulty [ASQ-MOTOR] at 18 months, β = −0.025 [95% CI, −0.035 to −0.015]; ASQ-MOTOR at 3 years, β = −0.029 [95% CI, −0.040 to −0.018]; and Child Development Inventory–Gross and Fine Motor Skills at 5 years, β = −0.028 [95% CI, −0.042 to −0.015]). Mendelian randomization analyses did not find any evidence for an association between maternal allele scores for birth weight and offspring neurodevelopmental difficulties.

**Conclusions and Relevance:**

This study found that the maternal intrauterine environment, as proxied by maternal birth weight genetic variants, is unlikely to be a major determinant of offspring neurodevelopmental outcomes.

## Key Points

**Question:**

Is the association between lower birth weight and offspring neurodevelopmental difficulties causal?

**Findings:**

In this conventional epidemiological cohort study of 46 970 offspring, lower birth weight was associated with neurodevelopmental difficulties across various offspring ages. However, mendelian randomization causal analyses of 44 134 mother-child dyads did not find evidence for a causal association between intrauterine growth (with maternal genetic factors influencing fetal growth as a proxy) and offspring neurodevelopmental difficulties.

**Meaning:**

This study found that maternal factors influencing intrauterine growth do not appear to drive the observational association between lower birth weight and offspring neurodevelopmental difficulties.

## Teaser

This cohort study investigates the association between lower birth weight and neurodevelopmental difficulties, as well as the causal association of intrauterine growth with measures of offspring neurodevelopmental difficulties.

## Introduction

The developmental origins of health and disease hypothesis proposes that the early developmental period, including intrauterine and early postnatal life, is critical for an individual’s long-term health. Among the pioneers^1^ of the hypothesis was David Barker, whose early work documented observational correlations between birth weight (as a proxy for fetal growth or development) and cardiometabolic health^2,3,4^; however, the hypothesis has since expanded to include other outcomes.^5,6^

Observational epidemiological studies suggest intrauterine growth restriction may lead to offspring neurodevelopmental difficulties (NDDs), including the development of language, motor, cognitive, and social skills; behavioral flexibility; and regulation of attention, activity, and impulses.^7,8,9^ Birth weight is a popular (yet imperfect) proxy for intrauterine growth that is associated with neurodevelopmental traits, including attention, working memory, executive function, global cognitive performance, and clinically diagnosed attention-deficit/hyperactivity disorder (ADHD) and autism.^10,11,12,13,14,15,16,17,18,19^

Most research on the developmental origins of health and disease is observational, and therefore associations between maternal pregnancy exposures and NDDs may reflect correlational rather than causal relationships. Many observational studies in this area have produced inconsistent or conflicting results,^6,8,9,20^ and even robust and replicated associations could be driven by latent confounding.^21^ Randomized clinical trials are considered the criterion standard for assessing causality but are often impractical or unethical, especially when maternal exposures during pregnancy and offspring outcomes are studied.^21^ Therefore, information regarding causality between developmental origins of health and disease exposures and offspring outcomes must primarily come from other study designs.

Mendelian randomization provides an alternative method to randomized clinical trials of investigating causal associations between an exposure and an outcome^22^ and uses genetic variants as instrumental variables to act as a proxy for an exposure of interest. Genetic variants, which are fixed at conception, are subject to Mendel’s laws of segregation and independent assortment and are therefore likely to be more robust to reverse causality and confounding than “traditional” epidemiological variables.^23^ Mendelian randomization has been used to investigate the causal association of maternal pregnancy exposures with offspring perinatal traits^24,25,26^ and later-life cardiometabolic risk factors.^26,27,28^ However, few mendelian randomization studies have examined the associations between maternal exposures and offspring NDDs,^29,30^ in part owing to the paucity of appropriately large genotyped family-based cohorts.^25^

**Figure 1.**
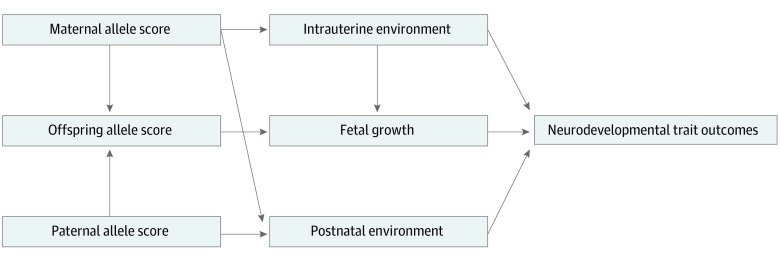
Diagram Illustrating the Potential Causal Associations Assessed With Maternal, Offspring, and Paternal Allele Scores for Birth Weight

To our knowledge, the Norwegian Mother, Father and Child Cohort Study (MoBa) is currently the world’s largest cohort of genotyped parent-offspring trios, containing maternal pregnancy exposure measurements and offspring neurodevelopmental measures from infancy to midchildhood. The availability of genotyped parents and offspring (as opposed to a cohort of unrelated individuals) allows for consistent estimates of the causal association of maternal exposures with offspring outcomes to be obtained via mendelian randomization.^31^ The present study used the mendelian randomization framework to assess whether intrauterine growth, using maternal genetic variants influencing offspring birth weight as a proxy, was causally associated with neurodevelopmental trait outcomes in up to 43 039 mother-offspring dyads available in MoBa (Figure 1).

## Methods

### Cohort Description

MoBa is a population-based pregnancy cohort study conducted by the Norwegian Institute of Public Health.^32^ Participants were recruited throughout Norway from June 1999 to December 2008. All pregnant women in Norway during this period were eligible for participation in MoBa, resulting in a total of 277 702 invitations sent.^32,33^ The women who consented to participate totaled 113 858 pregnancies (41%). Blood samples were obtained from both parents during pregnancy and from mothers and children (umbilical cord) at birth.^34^ The cohort includes approximately 114 500 children, 95 200 mothers, and 75 200 fathers. The present study is based on version 12 of the quality-assured data files released for research in January 2019. MoBa has been linked to the Medical Birth Registry of Norway, a national health registry containing information about all births in Norway. The establishment of MoBa and initial data collection was based on a license from the Norwegian Data Protection Agency and approval from the Regional Committees for Medical and Health Research Ethics. The MoBa cohort is currently regulated by the Norwegian Health Registry Act. The present study was approved by The Regional Committees for Medical and Health Research Ethics; written informed patient consent was obtained.

### Quality Control

**Figure 2.**
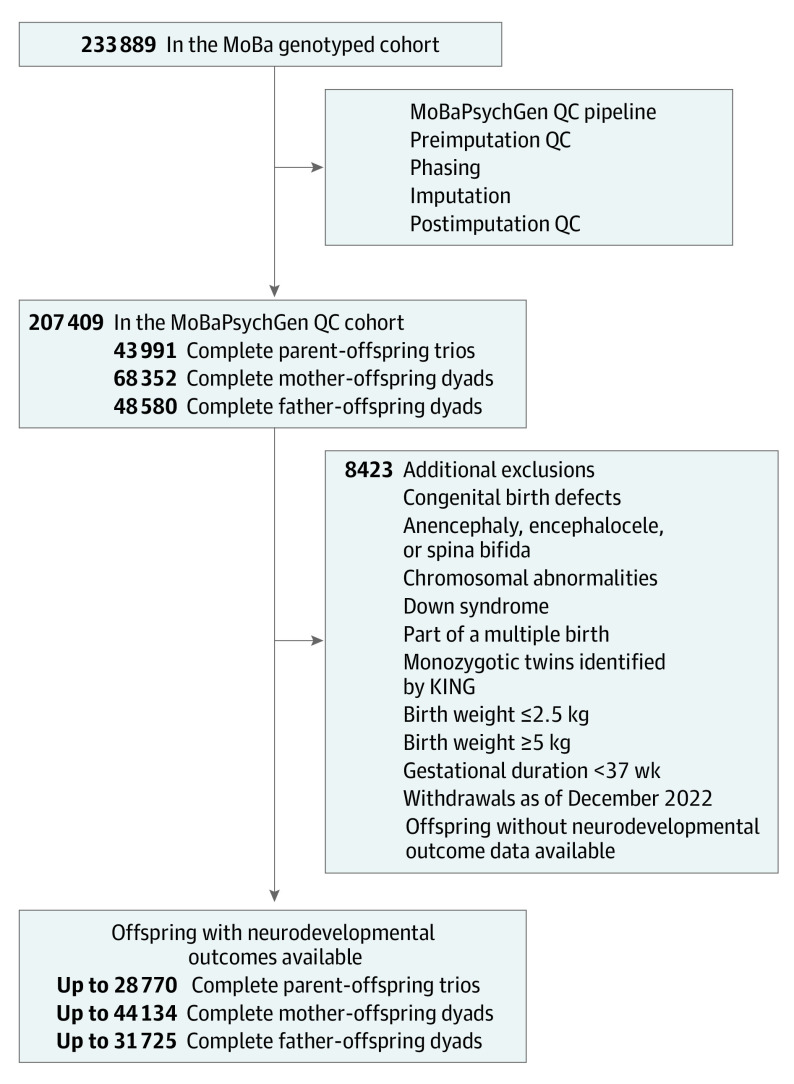
The Quality Control (QC) Process From the Norwegian Mother, Father and Child Cohort Study (MoBa) Genotyped Cohort to the Final Cohort Used in the Conventional Epidemiological and Mendelian Randomization Analyses MoBaPsychGen QC is described by Corfield et al.^35^ The total number of individuals in the MoBaPsychGen QC cohort includes families with 1 offspring, families with multiple offspring, and singletons. Additional exclusions related to the project at hand were conducted according to variables available in the Medical Birth Registry of Norway, as well as relatedness identified by KING in the MoBaPsychGen QC pipeline. Offspring were excluded if they had congenital birth defects, anencephaly, encephalocele, spina bifida, chromosomal abnormalities, or Down syndrome or were a part of a multiple birth. Offspring with a length of gestation fewer than 37 weeks (based on ultrasonographic examination or, if unavailable, date since last menstrual period) were excluded, as well as those with a birth weight less than or equal to 2.5 kg or greater than or equal to 5 kg. Gestational age and birth weight exclusions were performed to remove implausible measures and minimize the association of outliers and gestational age with the results, broadly consistent with past studies of birth weight.^26^ KING indicates kinship-based inference for genome-wide association studies.

This project used MoBa genetic data that were cleaned and imputed in accordance with the MoBaPsychGen pipeline.^35^ Quality control steps are summarized in Figure 2 (eAppendix 1 in Supplement 1). Only individuals of White European ancestry were included in the cleaned data set to minimize potential bias from unmodeled population substructure.

### Phenotypic Data

The Medical Birth Registry of Norway contains measures of offspring birth weight, gestational duration, sex, birth year, and both maternal and paternal age at birth. MoBa mothers completed ratings of offspring NDDs at age 6 months, 18 months, 3 years, 5 years, and 8 years of age (detailed descriptions in eAppendix 2 and eTable 1 in Supplement 1).

### Phenotype Extraction and Quality Control

Scale and item-level data sets were generated from MoBa questionnaires with the phenotools R package, version 0.2.2 (Center for Open Science).^36^ Missing phenotypes were mean imputed for individuals with at least 50% nonmissing items. The NDD outcomes were transformed with rank-inverse normal transformations (eAppendix 3 in Supplement 1). Birth weight was *z* score transformed separately for male and female offspring, consistent with what was performed in the genome-wide association study of birth weight by Warrington et al.^26^

### Conventional Epidemiological Analyses

We used genetic linear mixed models to examine the association between birth weight and NDDs while adjusting for sex, birth year, gestational duration, genotyping batch, and maternal and paternal age at birth. Both birth weight and the NDD outcomes were treated as continuous variables. Further details about these analyses are given in eAppendix 4 in Supplement 1.

### Genetic Variant Selection

**Figure 3.**
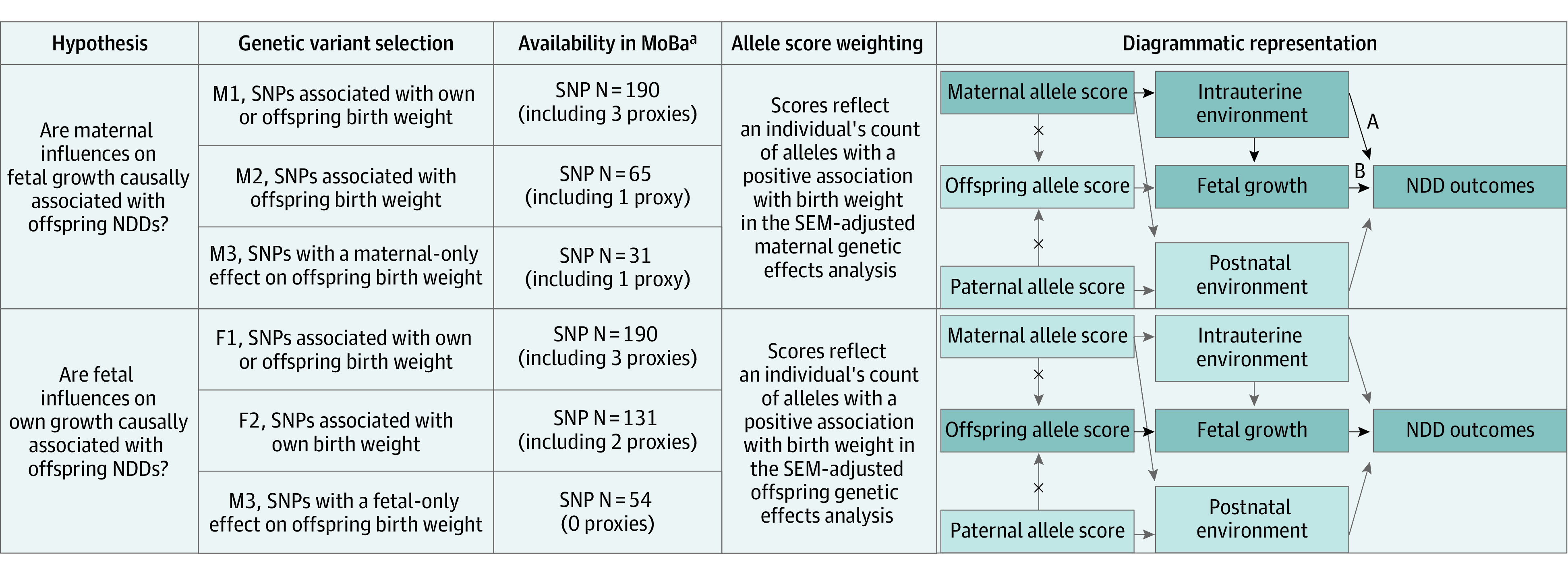
Details of the Allele Scoring Designed to Assess the Various Hypotheses in the Trio Analyses The black arrows and highlighted boxes represent the main causal pathways of interest. A total of 6 different unweighted allele scores were generated for mothers, fathers, and offspring in MoBa (maternal [M] 1, M2, and M3 and fetal [F] 1, F2, and F3). The scores designed to capture maternal influences on fetal growth (M1, M2, and M3) were weighted differently from the scores capturing direct fetal influences on fetal growth (F1, F2, and F3) (ie, based on the SEM in the study by Warrington et al^26^). In the primary analyses (M1, M2, and M3), we proposed using maternal allele scores as a proxy for the maternal intrauterine influences on fetal growth (ie, mechanisms related to the developmental origins of health and disease hypothesis) and included (1) SNPs associated with own or offspring birth weight (M1; SNP N = 205, of which 190 were available in MoBa after QC); (2) SNPs showing evidence of a maternal association with offspring birth weight (M2; SNP N = 71, of which 65 were available in MoBa after QC); and (3) SNPs with a maternal-only association with birth weight (M3; SNP N = 31, all available in MoBa after QC). Analyses were conditioned on offspring allele scores (to block the pathway from the fetus’ own genotype through to NDD outcomes via fetal growth) and paternal allele scores (to prevent possible introduction of collider bias). This model did not distinguish between the association between maternal allele score and the neurodevelopmental trait being mediated by fetal growth (path B) or due to a common intrauterine factors (path A). In addition, the availability of paternal allele scores provided a negative control in this framework. If there were an intrauterine effect, the maternal allele score–outcome association should be much stronger than the paternal allele score–outcome association.^37^ If maternal and paternal allele scores provide similar strengths of association, then it suggests the absence of intrauterine effects, and instead, any relationship is mediated by the postnatal environment.^37^ For the secondary analyses (F1, F2, and F3), offspring allele scores were used to test for a causal association between fetal growth and neurodevelopmental outcomes, which included SNPs associated with own or offspring birth weight (F1; SNP N = 205, of which 190 were available in MoBa after QC), SNPs showing evidence of a direct fetal genetic association with birth weight (F2; SNP N = 143, of which 131 were available in MoBa after QC), and SNPs shown to have a fetal-only association with birth weight (F3; SNP N = 63, of which 54 were available in MoBa after QC). Here the analyses were conditional on maternal allele scores (ie, to block maternal mechanisms mediated through the intrauterine or postnatal environment) and also the paternal allele scores (ie, to block postnatal paternal mechanisms). The SNPs in set 1 (either M1 or F1) were a superset of set 2 (M2 or F2), which in turn was a superset of set 3 (M3 or F3). Three different SNP sets were chosen because it was unclear a priori which set would best serve as a proxy for maternal and fetal influences on intrauterine growth because many birth weight–associated variants are known to exhibit both maternal and fetal effects.^26,38^ Even if a variant does not meet the criterion for statistical significance (ie, not significant in the SEM analysis as required for set M3), it may still act as a proxy for intrauterine growth and explain nontrivial proportions of the variance when combined (in sets M1 or M2) with other variants that also do not quite meet the threshold for significance. It is also possible that these SNPs are not truly associated with birth weight, in which case their addition to an allele score will tend to add noise. Only trio analyses are shown to illustrate the most robust study design (ie, allowing for blocking of pleiotropic pathways and accounting for possible correlation between maternal and paternal allele scores); however, additional better powered, although less stringent, maternal dyad analyses (and paternal dyad analyses) were also conducted with the same set of allele scores. MoBa indicates Norwegian Mother, Father and Child Cohort Study; NDD, neurodevelopmental difficulty; QC, quality control; SEM, structural equation model; and SNP, single-nucleotide polymorphism. ^a^The number of SNPs available in MoBa includes the proxy variants.

Autosomal single-nucleotide polymorphisms (SNPs) shown to have robust and independent associations with birth weight (*P* < 5 × 10^−8^; *r*^2^ < 0.01) were used as a proxy for intrauterine growth.^26^ Warrington et al^26^ resolved genetic effects on birth weight into maternal and fetal contributions by using structural equation modeling. Single-nucleotide polymorphisms with significant maternal effects are thought to act “indirectly” on birth weight through the maternal genome, whereas variants with fetal effects influence birth weight “directly” via the fetal genome. We leveraged this information to deliberately capture related, although distinctly different, exposures. Proponents of the developmental origins of health and disease hypothesis do not believe birth weight has a direct causal association with offspring outcomes; instead, they hypothesize that an adverse maternal intrauterine environment can influence offspring outcomes. We used maternal SNPs with maternal effects on birth weight as a proxy for an adverse maternal intrauterine environment. We also assessed whether fetal growth itself may have a causal association with NDDs through fetal SNPs with fetal effects on birth weight. Six (overlapping) sets of variants were taken forward for allele scoring, hereafter referred to as maternal (M) 1, M2, and M3 and fetal (F) 1, F2, and F3 (eTable 7 in Supplement 2 lists all included SNPs). A detailed explanation of SNP selection is given in Figure 3. Proxy variant selection for missing SNPs is described in eAppendix 5 and eTable 2 in Supplement 1.

### Allele Score Generation

Offspring, maternal, and paternal (unweighted) allele scores were calculated as the sum of either maternal or fetal effect alleles (0, 1, or 2) across SNPs^26^ (eTable 7 in Supplement 2). Weighted allele scores were derived for sensitivity analyses, in which the dosage at each locus was weighted by the maternal (M1, M2, and M3) or fetal (F1, F2, and F3) genetic effect in structural equation modeling analyses by Warrington et al.^26^ We focused on unweighted scores instead of weighted ones because we consider birth weight an imperfect marker of fetal growth for which we do not have appropriate effect size estimates (see Discussion). Missing genotype handling is described in eAppendix 6 in Supplement 1. Benchmarking checks were performed to investigate the validity of the allele scores for mendelian randomization analysis (eAppendix 7 in Supplement 1).

### Mendelian Randomization Analyses

Mendelian randomization analyses using the GCTA software tool, version 1.93.2 beta,^39^ were performed to investigate a potential causal association between maternal and fetal influences on intrauterine growth and offspring neurodevelopment.^40^ We did not perform traditional instrumental variable analysis nor the usual suite of mendelian randomization sensitivity analyses because we did not have estimates of the effect of maternal genotypes on the intrauterine environment. We had only estimates of the association between maternal SNPs and offspring birth weight. Advocates of the developmental origins of health and disease hypothesis argue against the notion that birth weight itself causes adverse offspring outcomes. Rather the theory is that there exist several aspects of the intrauterine environment that exert adverse effects on offspring outcomes (with birth weight being one imperfect proxy for intrauterine growth). Consequently, in traditional mendelian randomization analyses, SNPs as proxies for one of these latent processes would yield different estimates of the “causal” association between birth weight and NDDs than SNPs as proxies for other latent processes. Therefore, when investigating the developmental origins of health and disease using birth weight–associated SNPs, (1) it would be inappropriate to estimate a causal association using traditional instrumental variable methods, and (2) sensitivity analyses (which rely on consistent causal association estimates) cannot be performed. Instead, we contend that testing whether maternal birth weight–associated SNPs are also associated with offspring NDDs (conditional on offspring genotype at the same loci) is informative regarding causality (ie, testing only the causal null). The presence of such an association provides evidence that some aspect of the intrauterine environment (with maternal genotypes associated with offspring birth weight as a proxy) has causal associations with future offspring NDDs. These issues were discussed at length in previous work that used similar methods to investigate the association of intrauterine growth with offspring cardiometabolic disease risk.^28^

We analyzed 3 overlapping samples: parent-offspring trios (up to 28 770), mother-offspring dyads (up to 44 134), and father-offspring dyads (up to 31 725). Our primary analyses consisted of maternal dyads owing to the larger sample size; however, complete parent-offspring trios and paternal dyads were also studied as sensitivity analyses (see Discussion). Fathers were included to serve as a negative control (see Discussion). In the trio analyses, the fixed-effects part of the model included terms for the maternal, offspring, and paternal allele scores, as well as gestational age, sex, offspring birth year, maternal and paternal age at birth, and offspring genotyping batch, whereas the random-effects part of the model included an offspring genetic relationship matrix. The genetic relationship matrix was generated from all genotyped and imputed autosomal loci, excluding the birth weight variants (±1 megabase either side). Maternal dyad and paternal dyad analyses were similar except the allele score and covariates relating to the missing parent were not included. Sex-stratified analyses were also conducted. For weighted allele score sensitivity analyses, we adjusted for offspring-maternal-paternal dosages at each locus to block the pleiotropic path through the offspring-maternal-paternal genomes (where relevant). Instrument strength was assessed with the conditional *F* statistic (eAppendix 7 in Supplement 1).

We also performed principal component analysis to determine a multiple-testing–corrected *P* value threshold of *P* < .005 (eAppendix 8 and eTable 4 in Supplement 1); *P* values were 2-sided. We used R version 3.6.0 (R Foundation for Statistical Computing) for data analysis. Statistical power calculations for the mendelian randomization analyses are shown in eAppendix 9 in Supplement 1. This study followed the Strengthening the Reporting of Observational Studies in Epidemiology (STROBE) guidelines.

## Results

Descriptive statistics of the cohort after quality control are shown in eTable 5 in Supplement 1. The cohort after quality control had a mean (SD) maternal age at birth of 30.1 (4.5) years, mean (SD) paternal age at birth of 32.5 (5.1) years, and a median offspring birth year of 2005 (range, 1999-2009). There was no evidence of large differences in the mean values of the trio, maternal-dyad, and paternal-dyad data.

### Conventional Epidemiological Analyses

**Figure 4.**
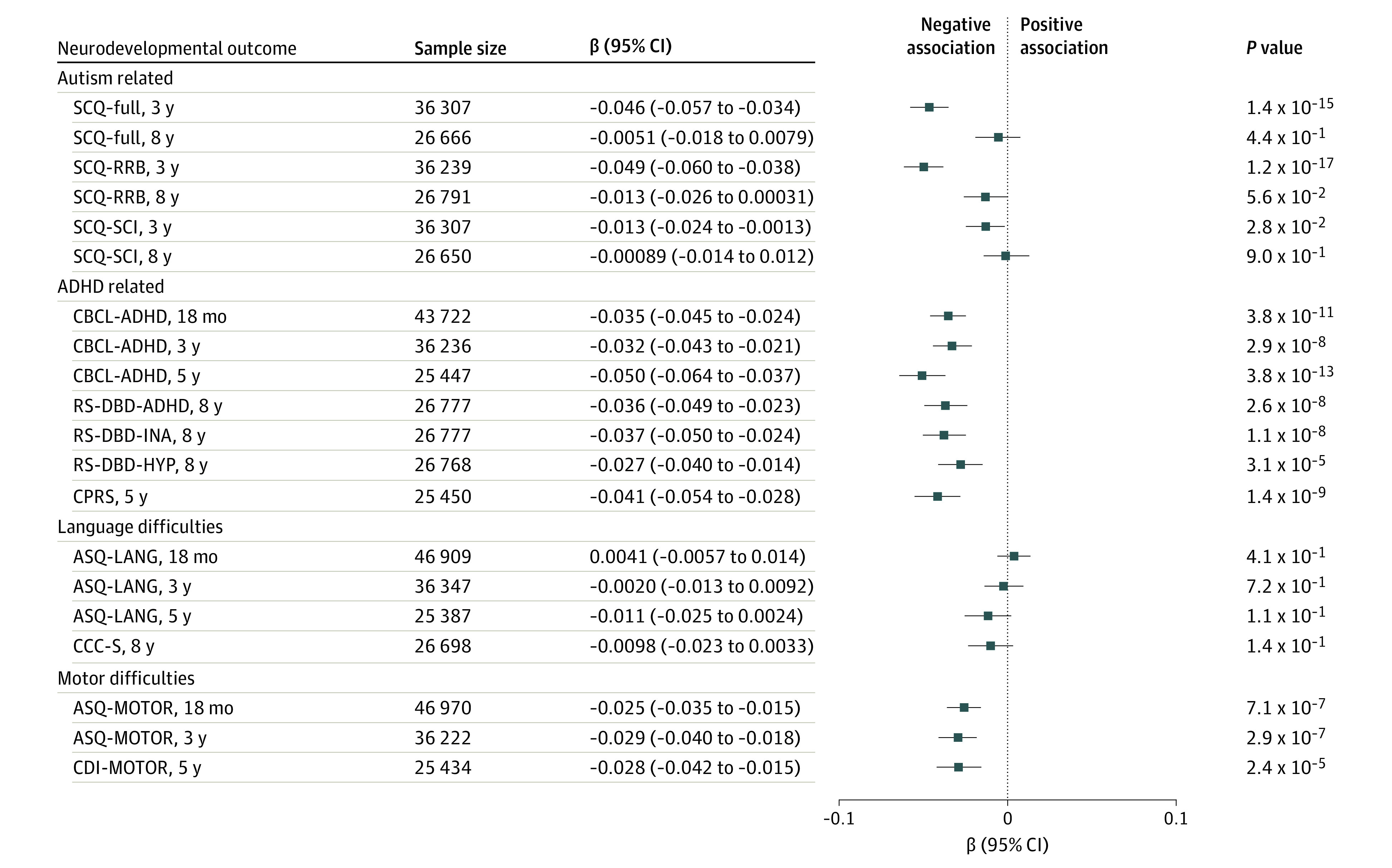
Conventional Epidemiological Associations Between Birth Weight and Neurodevelopmental Trait Outcomes While Adjusting for Covariates The model was adjusted for sex, birth year, maternal and paternal age at birth, gestational duration, and offspring genotyping batch and also included an offspring genetic relationship matrix. Neurodevelopmental difficulties were rated by mothers in the Norwegian Mother, Father and Child Cohort Study when the children were aged 18 months, 3 years, 5 years, and 8 years and included language and motor difficulties, inattention and hyperactivity-impulsivity, social communication difficulties, and repetitive behaviors. Estimates reflect standardized regression coefficients and 95% CIs. ASQ-LANG indicates Ages and Stages Questionnaire–Language; ASQ-MOTOR, ASQ–Motor Difficulty; CBCL-ADHD, Child Behavior Checklist–Attention Deficit Hyperactivity Disorder subscale; CCC-S, Children’s Communication Checklist–Short Scale for speech, language, or communication difficulties; CDI-MOTOR, Child Development Inventory–Gross and Fine Motor Skills; CPRS, Conners Parent Rating Scale–Revised (Short Form); RS-DBD-ADHD, Rating Scale for Disruptive Behavior Disorders–ADHD; RS-DBD-HYP, RS-DBD–Hyperactive-Impulsive Behavior; RS-DBD-INA, RS-DBD–Inattention; SCQ, Social Communication Questionnaire; SCQ-RRB, SCQ–Restricted and Repetitive Behaviors subscale; and SCQ-SCI, SCQ–Social Communication Impairments subscale.

The conventional epidemiological analyses of offspring (up to 46 970) found some evidence that birth weight was negatively associated with NDDs (Figure 4). Lower birth weight was significantly (*P* < .005) associated with increased autism-related trait scores (Social Communication Questionnaire [SCQ]–full at 3 years: β = −0.046 [95% CI, −0.57 to −0.034], *P* = 1.4 × 10^−15^; SCQ–Restricted and Repetitive Behaviors [RRB] at 3 years: β = −0.049 [95% CI, −0.060 to −0.038], *P* = 1.2 × 10^−17^), ADHD trait scores (Child Behavior Checklist [CBCL]–ADHD at 18 months: β = −0.035 [95% CI, −0.045 to −0.024], *P* = 3.8 × 10^−11^; CBCL-ADHD at 3 years: β = −0.032 [95% CI, −0.043 to −0.021], *P* = 2.9 × 10^−8^; CBCL-ADHD at 5 years: β = −0.050 [95% CI, −0.064 to −0.037], *P* = 3.8 × 10^−13^; Rating Scale for Disruptive Behavior Disorders [RS-DBD]–ADHD at 8 years: β = −0.036 [95% CI, −0.049 to −0.023], *P* = 2.6 × 10^−8^; RS-DBD–Inattention [INA] at 8 years: β = −0.037 [95% CI, −0.050 to −0.024], *P* = 1.1 × 10^−8^; RS-DBD–Hyperactive-Impulsive Behavior [HYP] at 8 years: β = −0.027 [95% CI, −0.040 to −0.014], *P* = 3.1 × 10^−5^; Conners Parent Rating Scale–Revised [Short Form] [CPRS] at 5 years: β = −0.041 [95% CI, −0.054 to −0.028], *P* = 1.4 × 10^−9^), and motor scores (Ages and Stages Questionnaire–Motor Difficulty [ASQ-MOTOR] at 18 months: β = −0.025 [95% CI, −0.035 to −0.015], *P* = 7.1 × 10^−7^; ASQ-MOTOR at 3 years: β = −0.029 [95% CI, −0.040 to −0.018], *P* = 2.9 × 10^−7^; and Child Development Inventory–Gross and Fine Motor Skills [CDI-MOTOR] at 5 years: β = −0.028 [95% CI, −0.042 to −0.015], *P* = 2.4 × 10^−5^), but not with any language-related traits. Results followed a similar trend when stratified by offspring sex (eAppendix 4 in Supplement 1).

### Mendelian Randomization Analyses

Our allele score benchmarking analyses demonstrated that offspring, maternal, and paternal scores were all significantly and positively associated with birth weight (eAppendix 7 in Supplement 1), and conditional *F* statistics suggest that our instruments were strong (*F* > 10) (eTable 3 in Supplement 1). Our allele score benchmarking analyses demonstrated that offspring scores (F1, F2, and F3) and maternal scores (M1, M2, and M3) were all significantly and positively associated with birth weight (M1: β = 0.006, 95% CI, 0.005-0.007; M2: β = 0.013, 95% CI, 0.011-0.015; M3: β = 0.018, 95% CI, 0.014-0.021; F1: β = 0.013, 95% CI, 0.011-0.014; F2: β = 0.018, 95% CI, 0.017-0.020; and F3: β = 0.020, 95% CI, 0.018-0.022). The primary analyses (ie, maternal dyads [up to 44 134 maternal dyads], maternal influences, and M1, M2, and M3) (Table) found little evidence for an association between maternal allele scores and offspring NDDs after adjusting for offspring scores (*P* < .05 for CBCL-ADHD at 18 months; β = −1.27E-03; SE = 6.02E-04). However, there was some evidence for associations between offspring (M1, M2, and M3) allele scores and NDDs after conditioning on maternal scores (Table) (*P* < .05 for CBCL-ADHD at 18 months: β = 1.24E-03, SE = 6.02E-04; CBCL-ADHD at 5 years: β = −1.51E-03, SE = 7.66E-04; ASQ-Language [LANG] at 5 years: β = −1.64E-03, SE = 6.56E-04; and CDI-MOTOR at 5 years: β = −1.34E-03, SE = 6.37E-04); however, these associations did not pass the more stringent multiple-testing correction threshold.

**Table.** Results From the Mendelian Randomization Analyses in Mother-Offspring Dyads^a^

Domain and outcome	No.	M1 or F1			M2 or F2			M3 or F3		
		Effect size estimate, β	SE	*P* value	Effect size estimate, β	SE	*P* value	Effect size estimate, β	SE	*P* value
**A. Maternal allele score (conditional on offspring allele score)**										
Difficulties with social communication and behavioral flexibility (repetitive behaviors)										
SCQ-full, 3 y	34 046	−1.58E-04	6.77E-04	.82	8.61E-04	1.24E-03	.49	−1.71E-04	1.92E-03	.93
SCQ-full, 8 y	24 968	−5.55E-04	7.65E-04	.47	−6.39E-04	1.39E-03	.65	8.49E-04	2.17E-03	.70
SCQ-RRB, 3 y	33 980	−1.85E-04	6.72E-04	.78	3.43E-04	1.23E-03	.78	−1.19E-03	1.91E-03	.53
SCQ-RRB, 8 y	25 091	4.14E-04	6.29E-04	.51	7.85E-04	1.14E-03	.49	2.57E-03	1.78E-03	.15
SCQ-SCI, 3 y	34 046	−6.20E-05	6.54E-04	.92	9.78E-04	1.20E-03	.41	8.15E-04	1.86E-03	.66
SCQ-SCI, 8 y	24 953	−8.94E-04	7.55E-04	.24	−1.31E-03	1.37E-03	.34	−5.43E-04	2.14E-03	.80
Difficulties with attention and hyperactive-impulsive behavior										
CBCL-ADHD, 18 mo	40 988	−1.27E-03	6.02E-04	.03	3.93E-04	1.10E-03	.72	1.78E-03	1.71E-03	.30
CBCL-ADHD, 3 y	33 980	−2.96E-04	6.73E-04	.66	2.69E-04	1.23E-03	.83	3.14E-03	1.91E-03	.10
CBCL-ADHD, 5 y	23 855	1.26E-03	7.67E-04	.10	1.14E-03	1.40E-03	.42	2.73E-04	2.17E-03	.90
RS-DBD-ADHD, 8 y	25 077	7.69E-04	7.66E-04	.32	1.70E-03	1.39E-03	.22	2.30E-03	2.17E-03	.29
RS-DBD-INA, 8 y	25 077	6.02E-04	7.58E-04	.43	1.46E-03	1.38E-03	.29	1.18E-03	2.15E-03	.58
RS-DBD-HYP, 8 y	25 068	5.49E-04	7.38E-04	.46	9.79E-04	1.34E-03	.47	1.81E-03	2.09E-03	.39
CPRS, 5 y	23 856	−5.43E-04	7.66E-04	.48	−2.04E-04	1.40E-03	.88	−2.09E-03	2.17E-03	.34
Difficulties with language										
ASQ-LANG, 18 mo	44 079	−3.58E-04	5.00E-04	.47	−1.12E-03	9.15E-04	.22	−2.63E-03	1.42E-03	.06
ASQ-LANG, 3 y	34 079	1.58E-04	5.43E-04	.77	4.20E-04	9.92E-04	.67	1.99E-03	1.54E-03	.20
ASQ-LANG, 5 y	23 798	1.08E-03	6.56E-04	.10	6.30E-04	1.20E-03	.60	3.09E-04	1.86E-03	.87
CCC-S, 8 y	24 999	−7.19E-04	7.71E-04	.35	−1.20E-03	1.40E-03	.39	−1.01E-03	2.18E-03	.64
Difficulties with motor skills										
ASQ-MOTOR, 18 mo	44 134	8.28E-04	4.68E-04	.08	1.07E-03	8.56E-04	.21	2.68E-04	1.33E-03	.84
ASQ-MOTOR, 3 y	33 961	−5.14E-04	5.82E-04	.38	−5.14E-04	1.06E-03	.63	6.59E-04	1.65E-03	.69
CDI-MOTOR, 5 y	23 843	7.36E-04	6.38E-04	.25	1.09E-03	1.17E-03	.35	8.22E-04	1.81E-03	.65
**B. Offspring allele score (conditional on maternal allele score)**										
Difficulties with social communication and behavioral flexibility (repetitive behaviors)										
SCQ-full, 3 y	34 046	1.88E-04	6.75E-04	.78	−9.81E-04	1.23E-03	.43	−4.11E-04	1.92E-03	.83
SCQ-full, 8 y	24 968	−4.73E-04	7.63E-04	.54	−1.30E-03	1.39E-03	.35	−1.04E-03	2.16E-03	.63
SCQ-RRB, 3 y	33 980	1.67E-04	6.69E-04	.80	−7.43E-04	1.22E-03	.54	1.13E-04	1.91E-03	.95
SCQ-RRB, 8 y	25 091	−1.08E-03	6.28E-04	.09	−1.66E-03	1.15E-03	.15	−2.21E-03	1.78E-03	.22
SCQ-SCI, 3 y	34 046	−4.10E-05	6.52E-04	.95	−1.06E-03	1.19E-03	.37	−3.75E-04	1.86E-03	.84
SCQ-SCI, 8 y	24 953	4.90E-05	7.54E-04	.95	−6.73E-04	1.38E-03	.63	8.70E-05	2.14E-03	.97
Difficulties with attention and hyperactive-impulsive behavior										
CBCL-ADHD, 18 mo	40 988	1.24E-03	6.02E-04	.04	7.30E-05	1.10E-03	.95	1.93E-04	1.70E-03	.91
CBCL-ADHD, 3 y	33 980	6.35E-04	6.71E-04	.34	2.11E-04	1.23E-03	.86	−1.74E-03	1.91E-03	.36
CBCL-ADHD, 5 y	23 855	−1.51E-03	7.66E-04	.05	−7.96E-04	1.40E-03	.57	−2.30E-03	2.17E-03	.29
RS-DBD-ADHD, 8 y	25 077	−7.44E-04	7.64E-04	.33	−1.36E-03	1.39E-03	.33	−5.65E-04	2.17E-03	.79
RS-DBD-INA, 8 y	25 077	−8.54E-04	7.57E-04	.26	−1.56E-03	1.38E-03	.26	−6.40E-05	2.15E-03	.98
RS-DBD-HYP, 8 y	25 068	−5.36E-04	7.36E-04	.47	−5.68E-04	1.34E-03	.67	−3.01E-04	2.09E-03	.88
CPRS, 5 y	23 856	−1.70E-04	7.65E-04	.82	−3.24E-04	1.40E-03	.82	1.82E-03	2.17E-03	.40
Difficulties with language										
ASQ-LANG, 18 mo	44 079	9.62E-04	4.99E-04	.05	1.29E-03	9.10E-04	.16	2.31E-03	1.42E-03	.10
ASQ-LANG, 3 y	34 079	−1.58E-04	5.42E-04	.77	−3.10E-04	9.90E-04	.75	−2.25E-03	1.54E-03	.14
ASQ-LANG, 5 y	23 798	−1.64E-03	6.55E-04	.01	−1.98E-03	1.20E-03	.10	3.06E-04	1.86E-03	.87
CCC-S, 8 y	24 999	4.23E-04	7.69E-04	.58	4.19E-04	1.40E-03	.76	1.49E-03	2.18E-03	.50
Difficulties with motor skills										
ASQ-MOTOR, 18 mo	44 134	−6.30E-05	4.67E-04	.89	−1.01E-03	8.52E-04	.24	−1.95E-03	1.33E-03	.14
ASQ-MOTOR, 3 y	33 961	5.10E-04	5.80E-04	.38	−2.00E-06	1.06E-03	>.99	1.27E-03	1.65E-03	.44
CDI-MOTOR, 5 y	23 843	−1.34E-03	6.37E-04	.04	−2.65E-03	1.17E-03	.02	−1.21E-03	1.80E-03	.50
**C. Offspring allele score (conditional on maternal allele score)**										
Difficulties with social communication and behavioral flexibility (repetitive behaviors)										
SCQ-full, 3 y	34 046	1.34E-03	6.71E-04	.04	9.81E-04	8.78E-04	.26	2.50E-04	1.30E-03	.85
SCQ-full, 8 y	24 968	1.39E-03	7.54E-04	.06	2.08E-03	9.85E-04	.03	2.40E-03	1.47E-03	.10
SCQ-RRB, 3 y	33 980	1.27E-03	6.65E-04	.06	1.24E-03	8.70E-04	.15	7.84E-04	1.29E-03	.54
SCQ-RRB, 8 y	25 091	−1.30E-05	6.21E-04	.98	−3.06E-04	8.11E-04	.71	3.01E-04	1.21E-03	.80
SCQ-SCI, 3 y	34 046	4.63E-04	6.48E-04	.48	−1.34E-04	8.48E-04	.87	−7.32E-04	1.25E-03	.56
SCQ-SCI, 8 y	24 953	1.48E-03	7.45E-04	.05	2.29E-03	9.73E-04	.02	2.28E-03	1.45E-03	.12
Difficulties with attention and hyperactive-impulsive behavior										
CBCL-ADHD, 18 mo	40 988	4.64E-04	5.97E-04	.44	2.53E-04	7.82E-04	.75	−8.97E-04	1.16E-03	.44
CBCL-ADHD, 3 y	33 980	4.19E-04	6.67E-04	.53	7.30E-05	8.72E-04	.93	1.21E-04	1.29E-03	.93
CBCL-ADHD, 5 y	23 855	−1.02E-03	7.55E-04	.18	−8.75E-04	9.94E-04	.38	3.09E-04	1.48E-03	.84
RS-DBD-ADHD, 8 y	25 077	−1.07E-03	7.55E-04	.16	−1.79E-03	9.86E-04	.07	−3.02E-03	1.47E-03	.04
RS-DBD-INA, 8 y	25 077	−7.97E-04	7.48E-04	.29	−1.29E-03	9.77E-04	.19	−1.64E-03	1.46E-03	.26
RS-DBD-HYP, 8 y	25 068	−1.21E-03	7.28E-04	.10	−2.11E-03	9.51E-04	.03	−3.43E-03	1.42E-03	.02
CPRS, 5 y	23 856	−2.22E-04	7.54E-04	.77	−6.30E-05	9.92E-04	.95	−1.44E-03	1.48E-03	.33
Difficulties with language										
ASQ-LANG, 18 mo	44 079	−5.80E-04	4.95E-04	.24	−1.04E-03	6.49E-04	.11	−1.23E-03	9.60E-04	.20
ASQ-LANG, 3 y	34 079	−2.65E-04	5.38E-04	.62	−1.54E-03	7.04E-04	.03	−1.98E-03	1.04E-03	.06
ASQ-LANG, 5 y	23 798	−4.05E-04	6.47E-04	.53	−3.80E-05	8.51E-04	.96	9.20E-04	1.27E-03	.47
CCC-S, 8 y	24 999	−5.38E-04	7.60E-04	.48	−1.07E-03	9.93E-04	.28	−2.10E-03	1.48E-03	.16
Difficulties with motor skills										
ASQ-MOTOR, 18 mo	44 134	1.85E-04	4.64E-04	.69	1.74E-04	6.08E-04	.78	−5.60E-04	8.99E-04	.53
ASQ-MOTOR, 3 y	33 961	2.55E-04	5.76E-04	.66	4.50E-05	7.53E-04	.95	−5.62E-04	1.12E-03	.61
CDI-MOTOR, 5 y	23 843	−3.27E-04	6.28E-04	.60	−7.70E-04	8.26E-04	.35	−2.14E-03	1.23E-03	.08
**D. Maternal allele score (conditional on offspring allele score)**										
Difficulties with social communication and behavioral flexibility (repetitive behaviors)										
SCQ-full, 3 y	34 046	−2.73E-04	6.70E-04	.68	5.20E-05	8.77E-04	.95	9.35E-04	1.30E-03	.47
SCQ-full, 8 y	24 968	−3.72E-04	7.54E-04	.62	−4.79E-04	9.90E-04	.63	−2.41E-04	1.48E-03	.87
SCQ-RRB, 3 y	33 980	1.56E-04	6.64E-04	.81	1.92E-04	8.70E-04	.83	7.16E-04	1.29E-03	.58
SCQ-RRB, 8 y	25 091	−2.44E-04	6.20E-04	.69	−5.53E-04	8.14E-04	.50	−2.00E-03	1.21E-03	.10
SCQ-SCI, 3 y	34 046	−7.07E-04	6.47E-04	.28	−2.93E-04	8.47E-04	.73	6.57E-04	1.26E-03	.60
SCQ-SCI, 8 y	24 953	−2.74E-04	7.44E-04	.71	−2.69E-04	9.78E-04	.78	8.17E-04	1.46E-03	.58
Difficulties with attention and hyperactive-impulsive behavior										
CBCL-ADHD, 18 mo	40 988	−4.97E-04	5.96E-04	.40	1.00E-04	7.81E-04	>.99	1.53E-03	1.15E-03	.18
CBCL-ADHD, 3 y	33 980	2.54E-04	6.66E-04	.70	1.49E-03	8.71E-04	.09	1.55E-03	1.29E-03	.23
CBCL-ADHD, 5 y	23 855	6.28E-04	7.58E-04	.41	3.18E-04	9.92E-04	.75	−2.05E-03	1.48E-03	.16
RS-DBD-ADHD, 8 y	25 077	9.16E-04	7.54E-04	.22	1.10E-03	9.91E-04	.27	1.25E-03	1.48E-03	.40
RS-DBD-INA, 8 y	25 077	1.11E-03	7.47E-04	.14	1.11E-03	9.81E-04	.26	1.21E-03	1.46E-03	.41
RS-DBD-HYP, 8 y	25 068	6.95E-04	7.27E-04	.34	1.07E-03	9.55E-04	.26	1.14E-03	1.42E-03	.42
CPRS, 5 y	23 856	5.80E-05	7.56E-04	.94	−6.11E-04	9.90E-04	.54	−6.21E-04	1.47E-03	.67
Difficulties with language										
ASQ-LANG, 18 mo	44 079	9.39E-04	4.95E-04	.06	8.96E-04	6.47E-04	.17	8.67E-04	9.58E-04	.36
ASQ-LANG, 3 y	34 079	−1.00E-05	5.37E-04	.98	5.32E-04	7.03E-04	.45	2.15E-04	1.04E-03	.84
ASQ-LANG, 5 y	23 798	6.40E-04	6.48E-04	.32	2.90E-05	8.49E-04	.97	−1.35E-03	1.26E-03	.29
CCC-S, 8 y	24 999	4.49E-04	7.59E-04	.55	7.86E-04	9.97E-04	.43	1.61E-03	1.49E-03	.28
Difficulties with motor skills										
ASQ-MOTOR, 18 mo	44 134	4.47E-04	4.64E-04	.34	1.13E-04	6.06E-04	.85	2.34E-04	8.97E-04	.79
ASQ-MOTOR, 3 y	33 961	−5.18E-04	5.75E-04	.37	−3.56E-04	7.53E-04	.64	−2.67E-04	1.12E-03	.81
CDI-MOTOR, 5 y	23 843	1.52E-04	6.29E-04	.81	−2.02E-04	8.24E-04	.81	3.56E-04	1.23E-03	.77

Abbreviations: ASQ, Ages and Stages Questionnaire; ASQ-LANG, ASQ-Language; ASQ-MOTOR, ASQ–Motor Difficulty; CBCL, Child Behavior Checklist; CBCL-ADHD, CBCL–Attention Deficit Hyperactivity Disorder subscale; CCC-S, Children’s Communication Checklist–Short Scale for speech, language, or communication difficulties; CDI-MOTOR, Child Development Inventory–Gross and Fine Motor Skills; CPRS, Conners Parent Rating Scale–Revised (Short Form); F, fetal; M, maternal; RS-DBD-ADHD, Rating Scale for Disruptive Behavior Disorders–ADHD; RS-DBD-HYP, RS-DBD–Hyperactive-Impulsive Behavior; RS-DBD-INA, RS-DBD–Inattention; SCQ, Social Communication Questionnaire; SCQ-RRB, SCQ–Restricted and Repetitive Behaviors subscale; SCQ-SCI, SCQ–Social Communication Impairments subscale.

^a^ GCTA-GREML was used to assess the relationship between maternal or offspring allele scores and offspring neurodevelopmental trait outcomes. Analyses were adjusted for offspring year of birth, maternal age at birth, offspring sex, and gestational duration; offspring genotyping batch parts A and B used maternal weighting (ie, M1, M2, and M3), and parts C and D used fetal weighting (F1, F2, and F3). Primary analyses used maternal allele M1, M2, and M3 scores (conditional on offspring M1, M2, and M3 scores), designed to capture mechanisms related to the developmental origins of health and disease hypothesis (part A). Secondary analyses used offspring F1, F2, and F3 allele scores (conditional on maternal F1, F2, and F3 scores), designed to capture fetal growth directly (part C). To assess whether a pleiotropic path may exist through the offspring genome, we also tested whether offspring M1, M2, and M3 scores were associated with offspring neurodevelopmental difficulty outcomes, conditional on maternal scores (part B). Likewise, we tested whether maternal F1, F2, and F3 scores were associated with offspring neurodevelopmental difficulty outcomes, conditional on fetal scores (part D).

Similarly, when allele scores with fetal influences were used (maternal dyads; F1, F2, and F3), there was some limited evidence for association between conditional offspring scores and NDDs (Table) (*P* < .05 for F1 and SCQ-full at 3 years: β = 1.34E-03, SE = 6.71E-04; F2 and SCQ-full at 8 years: β = 2.08E-03, SE = 9.85E-04; F1 and SCQ–Social Communication Impairments [SCI] at 8 years: β = 1.48E-03, SE = 7.45E-04; F2 and SCQ-SCI at 8 years: β = 2.29E-03, SE = 9.73E-04; F3 and RS-DBD-ADHD at 8 years: β = −3.02E-03, SE = 1.47E-03; F2 and RS-DBD-HYP at 8 years: β = −2.11E-03, SE = 9.51E-04; F3 and RS-DBD-HYP at 8 years: β = −3.43E-03, SE = 1.42E-03; and ASQ-LANG at 3 years: β = −1.54E-03, SE = 7.04E-04) and no evidence for association between conditional maternal scores and NDDs (Table).

Similar patterns of results were observed in the parent-offspring trio analyses (eTable 8 in Supplement 2 [ie, 2 nominally significant associations between maternal allele scores and NDDs]) (*P* < .05; M1 and SCQ-RRB at 8 years: β = 1.73E-03, SE = 8.08E-04; and M2 and SCQ-SCI at 8 years: β = 3.31E-03, SE = 1.46E-03), and there was evidence for association between offspring scores and NDDs (*P* < .05; M1 and SCQ-RRB at 8 years: β = −2.39E-03, SE = 9.32E-04; M2 and SCQ-RRB at 8 years: β = −5.17E-03, SE = 1.69E-03; M3 and SCQ-RRB at 8 years: β = −6.26E-03, SE = 2.62E-03; M1 and CBCL-ADHD at 5 years: β = −2.31E-03, SE = 1.12E-03; F3 and RS-DBD-ADHD at 8 years: β = −5.76E-03, SE = 2.15E-03; F3 and RS-DBD-INA at 8 years: β = −5.40E-03, SE = 2.13E-03; F3 and RS-DBD-HYP at 8 years: β = −4.27E-03, SE = 2.07E-03; and ASQ-LANG at 5 years: β = −1.90E-03, SE = 9.61E-04). Paternal allele scores, conditional on maternal and offspring scores, were also nominally associated with offspring NDDs (eTable 8 in Supplement 2) (*P* < .05; F1 and SCQ-full at 8 years: β = 2.06E-03, SE = 9.80E-04; M1 and SCQ-RRB at 8 years: β = 1.77E-03, SE = 8.12E-04; M2 and SCQ-RRB at 8 years: β = 4.60E-03, SE = 1.47E-03; M3 and SCQ-RRB at 8 years: β = 6.55E-03, SE = 2.25E-03; M3 and RS-DBD-ADHD at 8 years: β = 6.36E-03, SE = 2.76E-03; M3 and RS-DBD-INA at 8 years: β = 5.71E-03, SE = 2.73E-03; and M2 and ASQ-LANG at 3 years: β = 3.26E-03, SE = 1.28E-03).

Although not passing the strict multiple-testing correction threshold (*P* < .005), in the negative control analyses (ie, paternal dyads) (eTable 9 in Supplement 2), paternal allele scores showed a trend of positive associations with various offspring NDDs (*P* < .05; F1 and SCQ-full at 8 years: β = 1.99E-03, SE = 8.75E-04; M2 and SCQ-RRB at 8 years: β = 3.65E-03, SE = 1.32E-03; M3 and SCQ-RRB at 8 years: β = 5.50E-03, SE = 2.02E-03; F1 and SCQ-SCI at 8 years: β = 2.02E-03, SE = 8.64E-04; M2 and ASQ-LANG at 3 years: β = 2.05E-03, SE = 1.15E-03; and M2 and Children's Communication Checklist–Short Scale at 8 years: β = 3.35E-03, SE = 1.63E-03). In addition, offspring scores showed a trend of negative associations with offspring NDDs (*P* < .05; M1 and SCQ-RRB at 8 years: β = −1.56E-03, SE = 7.23E-04; M2 and SCQ-RRB at 8 years: β = −3.29E-03, SE = 1.32E-03; M3 and SCQ-RRB at 8 years: β = −4.12E-03, SE = 2.05E-03; F3 and RS-DBD-ADHD at 8 years: β = −4.96E-03, SE = 1.69E-03; F3 and RS-DBD-INA at 8 years: β = −4.12E-03, SE = 1.68E-03; F3 and RS-DBD-HYP at 8 years: β = −4.09E-03, SE = 1.63E-03; M1 and CPRS at 5 years: β = −1.85E-03, SE = 8.80E-04; F2 and ASQ-LANG at 18 months: β = −1.61E-03, SE = 7.66E-04; F3 and ASQ-LANG at 18 months: β = −2.48E-03, SE = 1.13E-03; F2 and ASQ-LANG at 3 years: β = −1.65E-03, SE = 8.14E-04; F3 and ASQ-LANG at 3 years: β = −2.50E-03, SE = 1.20E-03; and F3 and ASQ-MOTOR at 18 months: β = −2.43E-03, SE = 1.06E-03).

Sex-stratified analysis results are shown in eTables 10, 11, and 12 in Supplement 2. Weighted allele score analysis results are shown in eTables 13, 14, and 15 in Supplement 2.

## Discussion

Much of the previous research in the developmental origins of NDDs has been limited to conventional epidemiological studies, meaning that the causal nature of these associations is uncertain. Our conventional epidemiological analyses found that lower birth weight was significantly associated with many offspring NDDs across childhood, consistent with previous studies.^18,19,41^ However, these associations may be driven by latent confounding factors; therefore, we next used a more sophisticated causal inference approach.

This study used mendelian randomization principles to investigate the causal association of maternal influences on early growth with offspring NDDs after accounting for fetal effects. Despite being adequately powered to detect a modest maternal association, this study (consisting, to our knowledge, of the largest available source of genotyped trios) found no evidence of an association, suggesting that the maternal intrauterine environment (with SNPs with maternal associations with offspring birth weight as a proxy) may not be a major determinant of offspring NDDs. Instead, there was limited evidence that offspring allele scores were associated with many NDDs, even after conditioning on maternal (and paternal) scores. Together, these findings suggest that the conventional epidemiological association between low birth weight and offspring NDDs may be partially driven by genetic pleiotropy in the offspring genome rather than mechanisms of the developmental origins of health and disease, although these results did not survive multiple-testing correction and need to be replicated in an independent cohort.

In this study, we used SNPs associated with birth weight as a proxy for factors related to intrauterine growth. Our partitioning of genetic associations at birth weight–associated SNPs into maternal and fetal components allowed us to make inferences regarding causal effects of the intrauterine environment (ie, manifested as maternal associations of birth weight–associated SNPs with offspring neurodevelopment), causal effects of birth weight (ie, manifested as maternal and fetal associations of birth weight–associated SNPs with offspring neurodevelopment), or pleiotropy through the offspring genome (ie, manifested as fetal associations only of birth weight–associated SNPs with offspring neurodevelopment).

By including all 3 genotypes within the same model, we were able to determine the role of the maternal, paternal, and offspring genomes in mediating a potential causal association. Offspring genotypes are correlated with both maternal and paternal genotypes; therefore, failure to adjust for offspring genotypes can lead to violations of the mendelian randomization exclusion restriction assumption.^31,42^ Furthermore, modeling paternal genotypes will block any collider paths induced by conditioning on offspring allele score and can also act as a negative control. It is possible that selecting on the presence of both parents could introduce selection bias into the study if parental presence is associated with confounding factors. Although studying mother-child (or father-child) dyads attenuates this bias, the dyad study design may introduce collider bias. Therefore, by incorporating multiple designs (mother-child, father-child, and parent-offspring trio), we intended to mitigate the influence of such biases. By incorporating a genetic relationship matrix in the models, our approach should have been robust regarding the effects of population stratification and cryptic relatedness.

A negative control analysis was conducted to assess the association between paternal allele scores for birth weight and offspring NDDs.^43^ It is unlikely that these paternal SNPs can causally influence offspring neurodevelopment through intrauterine mechanisms; therefore, we did not expect to observe an association with offspring neurodevelopment unless driven by pleiotropic postnatal mechanisms or some other systematic bias.^37^ We found some evidence that paternal scores (conditional on offspring scores, maternal scores, or both) were positively associated with many offspring NDDs, although the paternal score was significant only after multiple-testing correction for SCQ-RRB score at 8 years in the parent-child trio analysis. One potential explanation for this unexpected observation is paternal selection bias in MoBa, based on paternal NDDs (eAppendix 11 and eTable 6 in Supplement 1). In addition, for the paternal-dyad analyses, most of the positive associations between paternal scores and offspring neurodevelopmental outcome were found when scores from SNPs that have maternal associations with birth weight were constructed (M1, M2, and M3), likely due to conditioning on a collider (ie, fetal genotypes).

### Limitations

An important limitation is that our study tests only that part of the intrauterine environment with maternal SNPs that are associated with offspring birth weight as a proxy. There may be other environmental exposures that are associated with the intrauterine environment for which we did not have SNP proxies, with causal associations with offspring NDDs. Consequently, additional mendelian randomization studies that have a range of different perinatal exposures as proxies are warranted (eg, gestational diabetes) to more fully investigate the association between early life exposures and offspring neurodevelopment. Our study serves as a useful framework for such future studies.

We developed 6 allele scores, the first set (M1, M2, and M3) intended to serve as a proxy for intrauterine exposures that affect offspring birth weight and the second set (F1, F2, and F3) designed to serve as a proxy for fetal growth directly through the fetal genome. However, we assumed that the partitioning of maternal and fetal genetic associations with birth weight was correct. In fact, our conditional allele score benchmarking analyses found that scores derived from SNPs with maternal-only associations with birth weight (eTable 3 in Supplement 1) (M3; β = 0.007 [95% CI, 0.003-0.010]; *P* = 3.10 × 10^−4^) still influence birth weight through the offspring genome (albeit weaker than the maternal association), meaning that scores (M1, M2, and M3) may not be serving solely as proxies for the maternal intrauterine environment as intended but may also capture fetal mechanisms. However, scores derived from fetal-only (F3) SNPs (eTable 3 in Supplement 1) did not appear to be associated with offspring birth weight through the maternal genome, supporting the original effect partitioning. Additionally, further investigation of maternal and fetal partitioning by Warrington et al^26^ using 13 934 MoBa maternal dyads found that the fetal variants explained 6% and maternal variants 2% of the variation in birth weight (genome-wide estimates of 28.5% and 7.6%), meaning that the maternal instrument is less powerful than the fetal one.^26^ There was also a small overlap between the data set used for instrument discovery and that used for estimation of causal associations, which can introduce a minor bias to the results (650 MoBa mothers in the maternal Early Growth Genetics genome-wide association study analysis, approximately half of whom would have responded to the questionnaires).^26,44^

We focused on unweighted allele scores because we consider birth weight to be an imperfect marker of latent intrauterine growth, for which we do not have appropriate effect size estimates.^28,42^ However, unweighted scores may disproportionately represent loci with small or large associations with birth weight and could cause issues in scores capturing both maternal and fetal mechanisms (M1, M2, F1, and F2 scores [eg, if acting in opposite directions]). Still, our weighted allele score sensitivity analyses found results similar to those of the unweighted analyses. There was some evidence that offspring M1-weighted scores were negatively associated with offspring SCQ-RRB scores at 8 years, conditional on maternal and paternal SNP dosages (eTable 13 in Supplement 2) (β = −0.15; *P* = .005). There was also a negative association between the conditional offspring F3-weighted score and RS-DBD-ADHD score at 8 years in paternal dyads (eTable 15 in Supplement 2) (β = −0.20; *P* = .002). However, we caution against overinterpretation of these results because the weightings reflect SNP associations with birth weight, not intrauterine growth.

Although our mendelian randomization results suggest that relatively small perturbations in intrauterine growth (with birth weight–associated SNPs as a proxy) may not play a large role in the development of NDDs in the general population, our paradigm may not be appropriate for estimating the causal association of more extreme environments because extreme environments (eg, famine, teratogen exposure, and severe infections during pregnancy) may not be captured by the genetic instrumental variables used in this study.^28^ Similarly, it is difficult when using mendelian randomization to examine associations at specific critical periods during pregnancy.^45^ Nevertheless, we believe our approach can inform the origin of the observational epidemiological association between birth weight and NDDs and that mendelian randomization should continue to be triangulated with other complementary approaches that rely on different assumptions. Last, this study focuses on measures of NDDs available in MoBa, rather than diagnoses in a smaller subset of the cohort, to increase statistical power and leverage the longitudinal information, which may be important because associations with neurodevelopment might manifest at specific developmental stages (eg, infancy, preschool age, and school age).

## Conclusion

In conclusion, this cohort study did not find any evidence that the maternal intrauterine environment, with genetic variants with maternal influences on offspring birth weight as a proxy, has a causal association with offspring NDDs but, instead, found some evidence that the conventional epidemiological associations between birth weight and offspring NDDs may be driven by genetic pleiotropy through the offspring genome. These results should be used to inform future research into the role of intrauterine growth on neurodevelopment.

## Notes

Supplement 1. **eAppendix 1.** Birthweight Distribution in MoBa **eFigure 1.** Histograms Displaying the Distribution of Birthweight in MoBa Offspring Before (Left) and After (Right) Additional Birthweight Exclusions Were Applied (< 2.5kg & > 5kg). **eAppendix 2.** Descriptions of Offspring Neurodevelopmental Difficulties **eTable 1.** Offspring Neurodevelopmental Outcomes. **eAppendix 3.** Offspring Neurodevelopmental Difficulty Phenotypic Transformations **eFigure 2**. Neurodevelopmental Trait Distributions Prior to Rank- Inverse Normal Transformation. **eFigure 3**. Neurodevelopmental Trait Distributions After Rank- Inverse Normal Transformation. **eAppendix 4.** Additional Information on Conventional Epidemiological Analyses **eFigure 4.** Sex-Stratified Conventional Epidemiological Associations Between Birthweight and Neurodevelopmental Trait Outcomes Whilst Adjusting for Covariates in Male Offspring. **eFigure 5.** Sex-Stratified Conventional Epidemiological Associations Between Birthweight and Neurodevelopmental Trait Outcomes Whilst Adjusting for Covariates in Female Offspring. **eFigure 6.** Conventional Epidemiological Associations Between Potential Confounders and Birthweight. **eAppendix 5.** Proxy SNP identification **eTable 2.** Genetic Variants Used to Proxy Missing Birthweight Variants in MoBa. **eAppendix 6.** Missing Genotype Handling **eAppendix 7.** SNP and Allele Score Benchmarking **eTable 3.** Association Between Offspring, Maternal and Paternal Birthweight Alleles Scores and Z-Transformed Birthweight. **eAppendix 8.** Principal Component Analysis **eTable 4**. Principal Components Analysis to Determine the Number of Independent Traits Assessed in the Present Study. **eAppendix 9.** Power Calculations **eFigure 7.** Maternal Genetic Effect Power Calculations for Mother-Child Dyad (N = 40,000) and Parent-Offspring Trio (N = 30,000) Analyses. **eFigure 8.** Offspring Genetic Effect Power Calculations for Mother-Child Dyad (N = 40,000) and Parent-Offspring Trio (N = 30,000) Analyses. **eFigure 9.** Maternal Genetic Effect Power Calculations for Mother-Child Dyad Analyses ((N = 40,000) Demonstrating the Power of the Study When Performing One Statistical Test Compared to 10 (Alpha = 0.05 and 0.005). **eTable 5**. Phenotypic and Genetic Characteristics of the MoBa Offspring, Mothers and Fathers after QC. **eAppendix 10.** Investigation Into Potential Paternal Selection Bias **eFigure 10.** Diagram Illustrating the Effect of Selection Bias on the Parent-Offspring Trio Study Design for Investigating the Effect of an Intrauterine Exposure (Proxied by Birthweight; BW) on Offspring Neurodevelopmental (ND) Outcomes. **eTable 6.** The Relationship Between Paternal Presence in MoBa and Offspring Neurodevelopmental Trait Outcomes.
eReferences.

Supplement 2. **eTable 7.** Genetic vAriants and Their Associations With Own and Offspring Birthweight in Warrington et al., 2019 and the Present Study **eTable 8.** Results From the Genetic Association Analyses in Parent-Offspring Trios **eTable 9.** Results From the Genetic Association Analyses in Father-Child Dyads **eTable 10.** Results From the Sex-Stratified Genetic Association Analyses in Parent-Offspring Trios **eTable 11.** Results From the Sex-Stratified Genetic Association Analyses in Mother-Child Dyads **eTable 12.** Results From the Sex-Stratified Genetic Association Analyses in Father-Child Dyads **eTable 13.** Results From the Weighted Allele Score Genetic Association Analyses in Parent-Offspring Trios **eTable 14.** Results From the Weighted Allele Score Genetic Association Analyses in Mother-Child Dyads **eTable 15.** Results From the Weighted Allele Score Genetic Association Analyses in Father-Child Dyads

Supplement 3.
Data Sharing Statement
