# Supplementary material for: Intrauterine Growth and Offspring Neurodevelopmental Traits: A Mendelian Randomization Analysis of the Norwegian Mother, Father and Child Cohort Study (MoBa)
Source: JAMA Psychiatry. 2023 Oct 25;81(2):144–56. doi: 10.1001/jamapsychiatry.2023.3872 (PMC10600722; doi:10.1001/jamapsychiatry.2023.3872)
Supplement: Supplement 1. — eAppendix 1. Birthweight Distribution in MoBa eFigure 1. Histograms Displaying the Distribution of Birthweight in MoBa Offspring Before (Left) and After (Right) Additional Birthweight Exclusions Were Applied (< 2.5kg & > 5kg). eAppendix 2. Descriptions of Offspring Neurodevelopmental Difficulties eTable 1. Offspring Neurodevelopmental Outcomes. eAppendix 3. Offspring Neurodevelopmental Difficulty Phenotypic Transformations eFigure 2. Neurodevelopmental Trait Distributions Prior to Rank- Inverse Normal Transformation. eFigure 3. Neurodevelopmental Trait Distributions After Rank- Inverse Normal Transformation. eAppendix 4. Additional Information on Conventional Epidemiological Analyses eFigure 4. Sex-Stratified Conventional Epidemiological Associations Between Birthweight and Neurodevelopmental Trait Outcomes Whilst Adjusting for Covariates in Male Offspring. eFigure 5. Sex-Stratified Conventional Epidemiological Associations Between Birthweight and Neurodevelopmental Trait Outcomes Whilst Adjusting for Covariates in Female Offspring. eFigure 6. Conventional Epidemiological Associations Between Potential Confounders and Birthweight. eAppendix 5. Proxy SNP identification eTable 2. Genetic Variants Used to Proxy Missing Birthweight Variants in MoBa. eAppendix 6. Missing Genotype Handling eAppendix 7. SNP and Allele Score Benchmarking eTable 3. Association Between Offspring, Maternal and Paternal Birthweight Alleles Scores and Z-Transformed Birthweight. eAppendix 8. Principal Component Analysis eTable 4. Principal Components Analysis to Determine the Number of Independent Traits Assessed in the Present Study. eAppendix 9. Power Calculations eFigure 7. Maternal Genetic Effect Power Calculations for Mother-Child Dyad (N = 40,000) and Parent-Offspring Trio (N = 30,000) Analyses. eFigure 8. Offspring Genetic Effect Power Calculations for Mother-Child Dyad (N = 40,000) and Parent-Offspring Trio (N = 30,000) Analyses. eFigure 9. Maternal Genetic Effect Power Calculations for M [file jamapsychiatry-e233872-s001.pdf]

Supplementary Online Content

D’Urso S, Moen GH, Hwang LD, et al. Intrauterine growth and offspring neurodevelopmental traits: a mendelian randomization analysis of the Norwegian Mother, Father and Child Cohort Study (MoBa). *JAMA Psychiatry*. Published online October 25, 2023. doi:10.1001/jamapsychiatry.2023.3872

eAppendix 1. Birthweight Distribution in MoBa
eFigure 1. Histograms Displaying the Distribution of Birthweight in MoBa Offspring Before (Left) and After (Right) Additional Birthweight Exclusions Were Applied (< 2.5kg & > 5kg).
eAppendix 2. Descriptions of Offspring Neurodevelopmental Difficulties
eTable 1. Offspring Neurodevelopmental Outcomes.
eAppendix 3. Offspring Neurodevelopmental Difficulty Phenotypic Transformations
eFigure 2. Neurodevelopmental Trait Distributions Prior to Rank- Inverse Normal Transformation.
eFigure 3. Neurodevelopmental Trait Distributions After Rank- Inverse Normal Transformation.
eAppendix 4. Additional Information on Conventional Epidemiological Analyses
eFigure 4. Sex-Stratified Conventional Epidemiological Associations Between Birthweight and Neurodevelopmental Trait Outcomes Whilst Adjusting for Covariates in Male Offspring.
eFigure 5. Sex-Stratified Conventional Epidemiological Associations Between Birthweight and Neurodevelopmental Trait Outcomes Whilst Adjusting for Covariates in Female Offspring.
eFigure 6. Conventional Epidemiological Associations Between Potential Confounders and Birthweight.
eAppendix 5. Proxy SNP identification
eTable 2. Genetic Variants Used to Proxy Missing Birthweight Variants in MoBa.
eAppendix 6. Missing Genotype Handling
eAppendix 7. SNP and Allele Score Benchmarking
eTable 3. Association Between Offspring, Maternal and Paternal Birthweight Alleles Scores and Z-Transformed Birthweight.
eAppendix 8. Principal Component Analysis
eTable 4. Principal Components Analysis to Determine the Number of Independent Traits Assessed in the Present Study.
eAppendix 9. Power Calculations
eFigure 7. Maternal Genetic Effect Power Calculations for Mother-Child Dyad (N = 40,000) and Parent-Offspring Trio (N = 30,000) Analyses.
eFigure 8. Offspring Genetic Effect Power Calculations for Mother-Child Dyad (N = 40,000) and Parent-Offspring Trio (N = 30,000) Analyses.
eFigure 9. Maternal Genetic Effect Power Calculations for Mother-Child Dyad Analyses ((N = 40,000) Demonstrating the Power of the Study When Performing One Statistical Test Compared to 10 (Alpha = 0.05 and 0.005).
eTable 5. Phenotypic and Genetic Characteristics of the MoBa Offspring, Mothers and Fathers after QC.
eAppendix 10. Investigation Into Potential Paternal Selection Bias
eFigure 10. Diagram Illustrating the Effect of Selection Bias on the Parent-Offspring Trio Study Design for Investigating the Effect of an Intrauterine Exposure (Proxied by Birthweight; BW) on Offspring Neurodevelopmental (ND) Outcomes.
eTable 6. The Relationship Between Paternal Presence in MoBa and Offspring Neurodevelopmental Trait Outcomes.
eReferences.

This supplementary material has been provided by the authors to give readers additional information about their work.

© 2023 D’Urso S et al. *JAMA Psychiatry*.

eAppendix 1. Birthweight distribution in MoBa

This project used MoBa genetic data that was cleaned and imputed as per the MoBaPsychGen pipeline[36]. In addition, offspring were excluded if they had congenital birth defects, anencephaly, encephalocele, spina bifida, chromosomal abnormalities, Down's syndrome, or were a part of a multiple birth. Offspring with a length of gestation less than 37 weeks (based upon ultrasound examination, or if unavailable, date since last menstrual period) were excluded, as well as those with a birthweight ≤ 2.5 kilograms or ≥ 5 kilograms. eFigure 1 displays the distribution of birthweight before and after exclusions were applied. Gestational age and birthweight exclusions were performed to remove implausible measures and minimise the influence of outliers and the effect of gestational age on the results, broadly consistent with past studies of birthweight[27]. Withdrawals from MoBa as of December 2022 were also excluded.

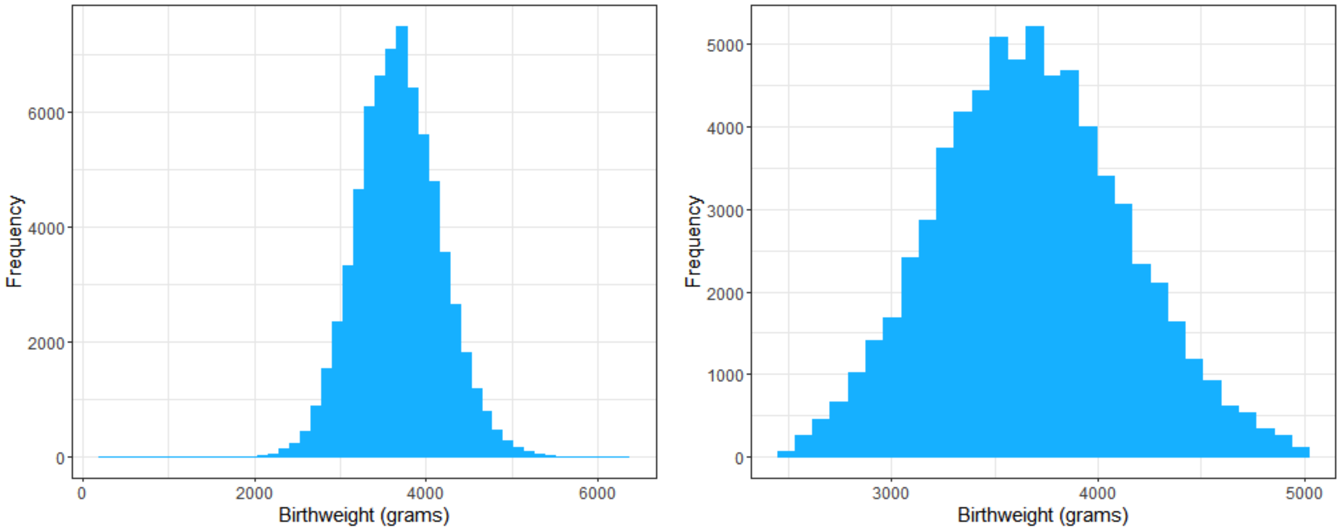

eFigure 1. Histograms displaying the distribution of birthweight in MoBa offspring before (left) and after (right) additional birthweight exclusions were applied (< 2.5kg & > 5kg).

Individuals in both panels passed all other exclusions (i.e., no reports of congenital birth defects, anencephaly, encephalocele, spina bifida, chromosomal abnormalities, Down's syndrome, multiple birth, twins, gestational length outliers, withdrawals).

© 2023 D'Urso S et al. *JAMA Psychiatry*.

eAppendix 2. Descriptions of offspring neurodevelopmental difficulties

Social Communication Questionnaire (SCQ)[37]. The SCQ is a parental-report questionnaire designed to identify signs of autism, which was completed by MoBa mothers when the child was 3 and 8 years of age[37]. At both ages, the SCQ consists of the same 40 items (yes/no response format with some items reverse-coded) relating to social and communication impairments (SCI) and restricted and repetitive behaviours (RRB)[37]. Higher scores are indicative of atypical behaviour (i.e., scored as 1 for atypical behavior and 0 for absence of atypical behavior/typical behavior[37].

Child Behaviour Checklist (CBCL)[38]. The CBCL was developed to identify problem behaviour in children[38]. The Diagnostic and Statistical Manual of Mental Disorders (DSM) oriented subscale for ADHD behaviors was used at age 18 months, 3 years and 5 years, including 4, 6 and 6 items, respectively. The items were rated by the mothers on a 3-point scale (0 = not true; 1 = somewhat or sometimes true; 2 = very true or often true), where higher scores indicate more ADHD-associated behaviors[38].

Rating Scale for Disruptive Behaviour Disorders (RS-DBD)[39]. The RS-DBD is based upon DSM-IV items and consists of 34 items relating to Oppositional Defiant Disorder, Conduct Disorder and ADHD. The 18 items related to ADHD were further divided into the subscales of inattention (9 items) and hyperactivity (9 items). Mothers evaluated their child's behaviour at age 8 years on a 4-point scale (0 = never or seldom; 1 = sometimes; 2 = often; 3 = very often), where high scores reflect more inattentive/hyperactive behaviours.

Conners Parent Rating Scale-Revised (Short Form) (CPRS-R (S))[40]. The CPRS-R (S) is a questionnaire used to obtain parental reports of childhood behaviour problems. The version administered in the MoBa questionnaire [39] at child age 5 years consists of 12 items relating to inattention, hyperactivity and impulsivity. Responses were scored on a 4-point Likert scale (0 = not true/never/seldom; 1 = somewhat true/sometimes; 2 = quite often; 3 = very often), where increased scores represent increased behavioural problems.

Ages and Stages Questionnaire (ASQ)[41–43]. The ASQ is part of an age-specific series of questionnaires for child development, with subscales relating to communication, gross motor, fine motor, problem solving and personal-social. The ASQ was included in the 18 months, 3 years and 5 years MoBa Questionnaires and parents answer 'yes', 'sometimes' or 'not yet'.

The Children's Communication Checklist-2 Short Scale (CCC-S)[44,45]. The CCC-S is a 13-item short scale of the CCC-2 that can identify children with potential speech, language and communication needs. Parents answer 'never or rarely', 'sometimes', 'often' or 'very often'.

Child Development Inventory (CDI)[46,47]. The CDI was designed to assess childhood development from ages 15 months to 6 years, and was included in the 5-year MoBa Questionnaire. This gross- and fine motor skill subscale consists of 12 yes/no questions, 10 of which are derived from the 1992 iteration of the CDI, while the other two items were designed specifically for MoBa. The CDI was reverse coded so that increased scores reflect greater motor difficulties.

Descriptions are summarised in eTable 1.

© 2023 D'Urso S et al. *JAMA Psychiatry*.

eTable 1. Offspring neurodevelopmental outcomes.

Domain	Measure	Subscales	Age Assessed (years)
Difficulties with social communication and behavioral flexibility (repetitive behaviors)	SCQ: Social Communication Questionnaire	Total	3
			8
		Restrictive and repetitive behaviours	3
			8
		Social communication difficulties	3
			8
Inattention and hyperactive-impulsive behavior	CBCL: Child Behaviour Checklist	ADHD: Total	1.5
			3
			5
	RS-DBD: Rating Scale for Disruptive Behaviour Disorders Attention deficit hyperactivity items	ADHD: Total	8
		ADHD: Inattention	8
		ADHD: Hyperactive-impulsive behaviour	8
	CPRS-R: Conners Parent Rating Scale-Revised (Short Form)	ADHD: Total	5
Language difficulties	ASQ: Ages and Stages Questionnaire	Language difficulties	1.5
			3
			5
	CCC-S: Children's Communication Checklist – Short Scale	Short Scale: Speech, language or communication difficulties	8
Motor difficulties	ASQ: Ages and Stages Questionnaire	Motor difficulties	1.5
			3
	CDI: Child Development Inventory	Gross- and Fine Motor Skills (reverse coded)	5

ADHD = attention deficit hyperactivity disorder.

© 2023 D'Urso S et al. *JAMA Psychiatry*.

eAppendix 3. Offspring neurodevelopmental difficulty phenotypic transformations

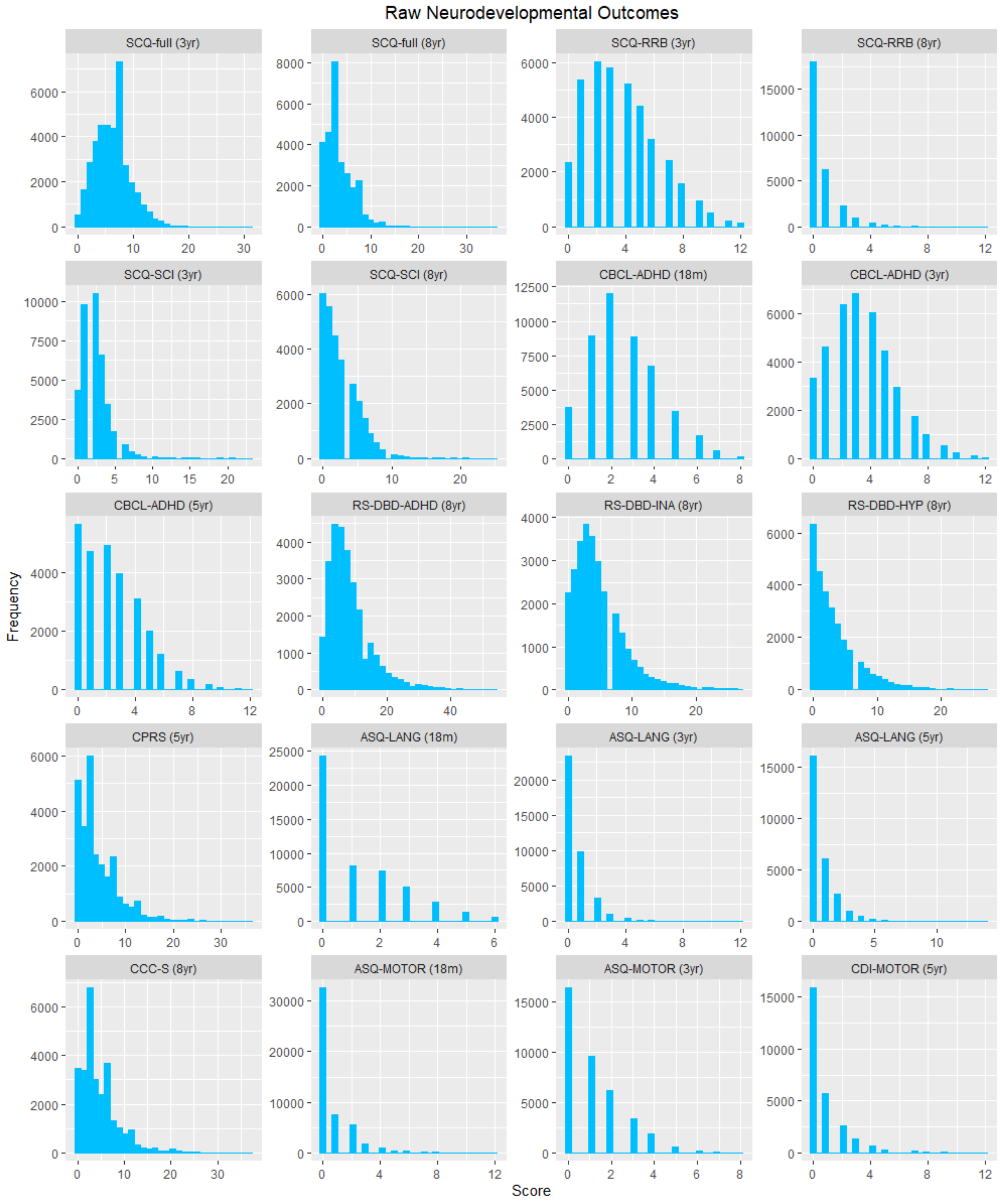

eFigure 2. Neurodevelopmental trait distributions prior to rank- inverse normal transformation.

© 2023 D'Urso S et al. *JAMA Psychiatry*.

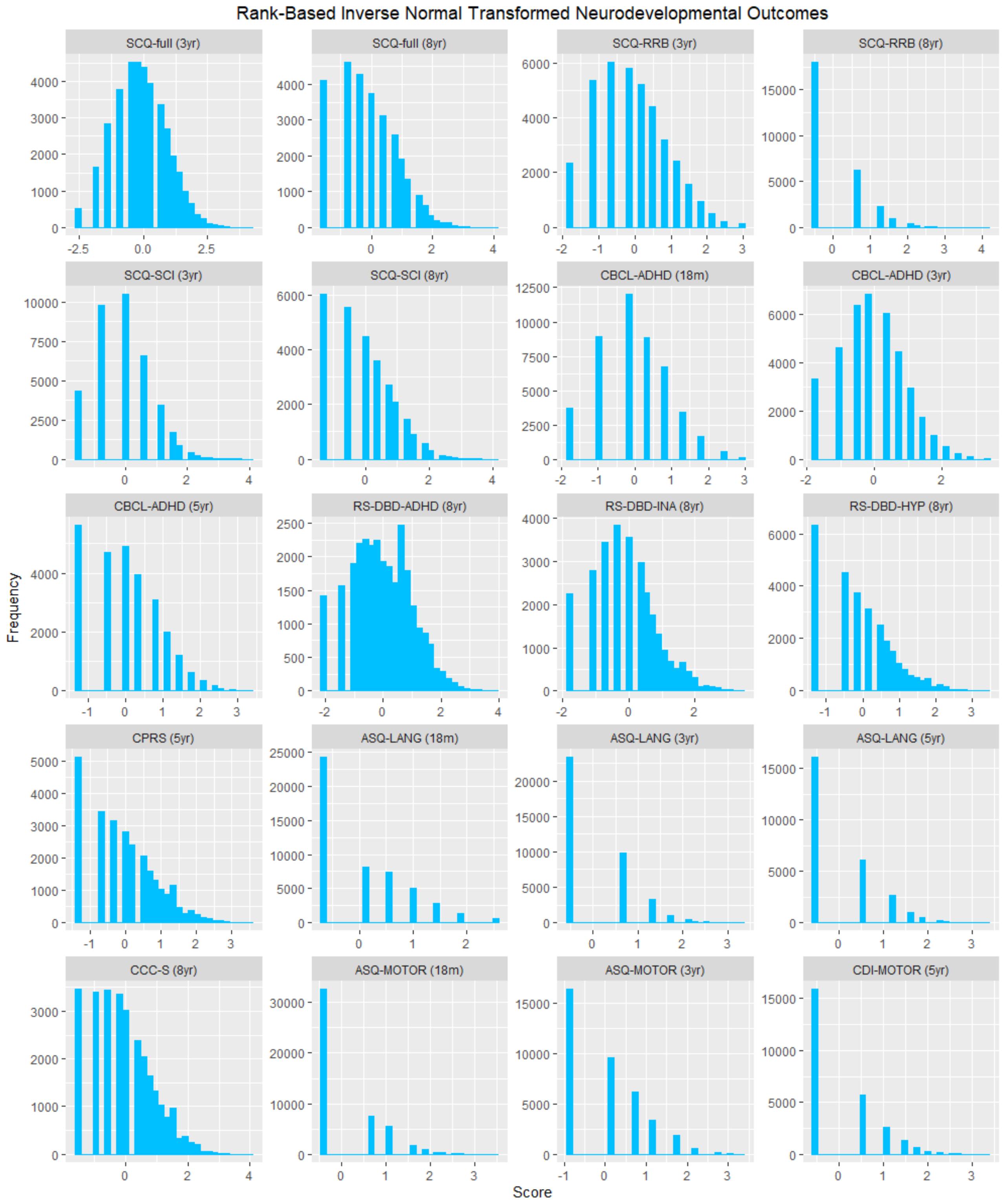

eFigure 3. Neurodevelopmental trait distributions after rank- inverse normal transformation.

© 2023 D'Urso S et al. *JAMA Psychiatry*.

eAppendix 4. Additional information on conventional epidemiological analyses

Conventional epidemiological analyses were conducted using the GCTA software tool v 1.93.2 beta[48,49]. This software implements a genetic linear mixed model where covariates and tests for association are fit in the fixed-effects part of the model, whereas cryptic relatedness and residual population stratification are modelled in the random-effects part of the model using a genetic relationship matrix (GRM), generated from all genotyped and imputed autosomal loci. The fixed-effects part of the model included terms for offspring sex, offspring birth year, maternal age at birth, paternal age at birth, gestational duration, and offspring genotyping batch, while the random-effects part of the model included a GRM of the offspring. Sex-stratified conventional epidemiological analyses were also conducted (eFigure 4 and eFigure 5).

To inform on which covariates should be included in the analyses described above, univariate linear regression analyses, were used to investigate the relationship between possible confounders (i.e., maternal and paternal age at birth, offspring birth year, gestational duration, trio membership, mother-child dyad membership and father-child dyad membership) and birthweight (conducted in R version 3.6.0). The relationship between birthweight and potential confounders are shown in eFigure 6.

Relationship between birthweight and neurodevelopmental trait outcomes in males only

Neurodevelopmental Outcomes (Autism-related)	Sample Size	Estimate	P-Value
SCQ-full (3yr)	18,515	-0.053	1.8×10^{-10}
SCQ-full (8yr)	13,575	-0.018	6.7×10^{-2}
SCQ-RRB (3yr)	18,480	-0.052	2.3×10^{-10}
SCQ-RRB (8yr)	13,639	-0.026	1.2×10^{-2}
SCQ-SCI (3yr)	18,518	-0.02	2.1×10^{-2}
SCQ-SCI (8yr)	13,567	-0.011	2.8×10^{-1}
**Neurodevelopmental Outcomes (ADHD-related)**	**Sample Size**	**Estimate**	**P-Value**
CBCL-ADHD (18m)	22,303	-0.033	1.1×10^{-5}
CBCL-ADHD (3yr)	18,489	-0.03	1.5×10^{-4}
CBCL-ADHD (5yr)	12,896	-0.062	5.1×10^{-10}
RS-DBD-ADHD (8yr)	13,625	-0.041	2.3×10^{-5}
RS-DBD-INA (8yr)	13,627	-0.043	1.1×10^{-5}
RS-DBD-HYP (8yr)	13,622	-0.032	1.2×10^{-3}
CPRS (5yr)	12,893	-0.049	1.4×10^{-6}
**Neurodevelopmental Outcomes (Language difficulties)**	**Sample Size**	**Estimate**	**P-Value**
ASQ-LANG (18m)	23,931	0.0044	5.6×10^{-1}
ASQ-LANG (3yr)	18,533	-0.0037	6.9×10^{-1}
ASQ-LANG (5yr)	12,857	-0.016	1.2×10^{-1}
CCC-S (8yr)	13,583	-0.015	1.2×10^{-1}
**Neurodevelopmental Outcomes (Motor difficulties)**	**Sample Size**	**Estimate**	**P-Value**
ASQ-MOTOR (18m)	23,970	-0.021	2.1×10^{-3}
ASQ-MOTOR (3yr)	18,458	-0.038	1.2×10^{-5}
CDI-MOTOR (5yr)	12,881	-0.033	3.0×10^{-3}

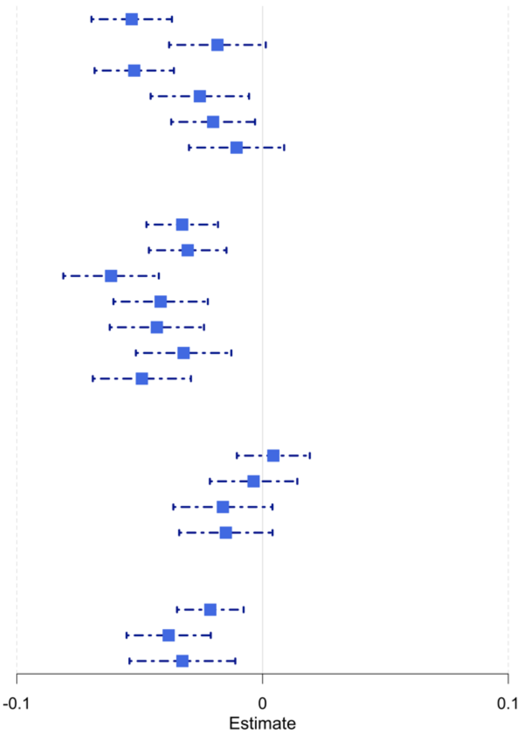

eFigure 4. Sex-stratified conventional epidemiological associations between birthweight and neurodevelopmental trait outcomes whilst adjusting for covariates in male offspring.

The model was adjusted for birth year, maternal and paternal age at birth, gestational duration, and offspring genotyping batch and included an offspring genetic relationship matrix. Estimates reflect standardised regression coefficients and 95% confidence intervals.

© 2023 D'Urso S et al. *JAMA Psychiatry*.

Relationship between birthweight and neurodevelopmental trait outcomes in females only

Neurodevelopmental Outcomes (Autism-related)	Sample Size	Estimate	P-Value
SCQ-full (3yr)	17,792	-0.038	8.1×10^{-7}
SCQ-full (8yr)	13,091	0.0077	3.8×10^{-1}
SCQ-RRB (3yr)	17,759	-0.046	7.5×10^{-9}
SCQ-RRB (8yr)	13,152	-0.00062	9.4×10^{-1}
SCQ-SCI (3yr)	17,789	-0.0052	4.9×10^{-1}
SCQ-SCI (8yr)	13,082	0.009	3.2×10^{-1}
**Neurodevelopmental Outcomes (ADHD-related)**	**Sample Size**	**Estimate**	**P-Value**
CBCL-ADHD (18m)	21,420	-0.038	3.6×10^{-7}
CBCL-ADHD (3yr)	17,747	-0.035	2.1×10^{-5}
CBCL-ADHD (5yr)	12,551	-0.039	4.9×10^{-5}
RS-DBD-ADHD (8yr)	13,152	-0.032	1.4×10^{-4}
RS-DBD-INA (8yr)	13,150	-0.032	1.5×10^{-4}
RS-DBD-HYP (8yr)	12,146	-0.024	5.2×10^{-3}
CPRS (5yr)	12,557	-0.033	2.4×10^{-4}
**Neurodevelopmental Outcomes (Language difficulties)**	**Sample Size**	**Estimate**	**P-Value**
ASQ-LANG (18m)	22,978	0.0031	6.2×10^{-1}
ASQ-LANG (3yr)	17,814	-0.0013	8.5×10^{-1}
ASQ-LANG (5yr)	12,530	-0.0066	4.7×10^{-1}
CCC-S (8yr)	13,115	-0.0049	5.9×10^{-1}
**Neurodevelopmental Outcomes (Motor difficulties)**	**Sample Size**	**Estimate**	**P-Value**
ASQ-MOTOR (18m)	23,000	-0.03	5.3×10^{-5}
ASQ-MOTOR (3yr)	17,764	-0.02	4.4×10^{-3}
CDI-MOTOR (5yr)	12,554	-0.022	3.4×10^{-3}

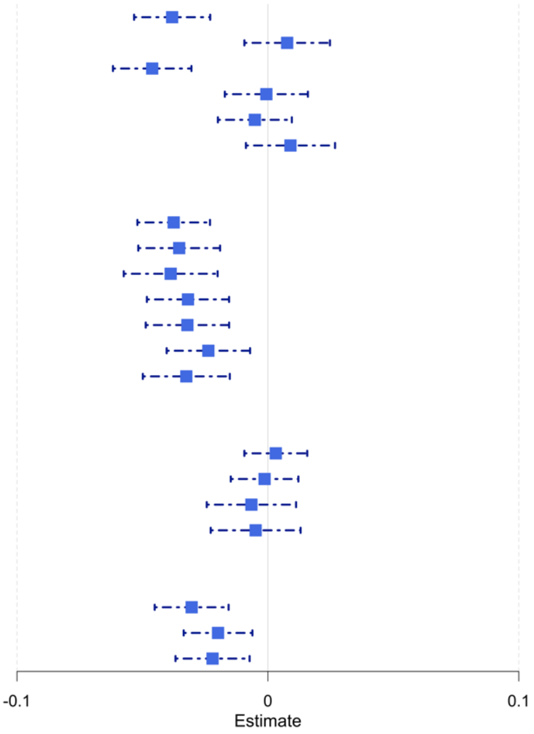

eFigure 5. Sex-stratified conventional epidemiological associations between birthweight and neurodevelopmental trait outcomes whilst adjusting for covariates in female offspring.
The model was adjusted for birth year, maternal and paternal age at birth, gestational duration, and offspring genotyping batch, and included an offspring genetic relationship matrix. Estimates reflect standardised regression coefficients and 95% confidence intervals.

Potential Confounder	Sample Size	Estimate	P-Value
Maternal age at birth	68,138	0.048	2.96×10^{-36}
Paternal age at birth	68,007	0.034	1.62×10^{-18}
Offspring birth year	68,142	-0.05	6.70×10^{-39}
Trio membership	68,142	-0.016	3.42×10^{-2}
Maternal-duo membership	68,142	-0.01	4.13×10^{-1}
Paternal-duo membership	68,142	-0.014	7.38×10^{-2}
Gestational duration (weeks)	67,929	0.39	0

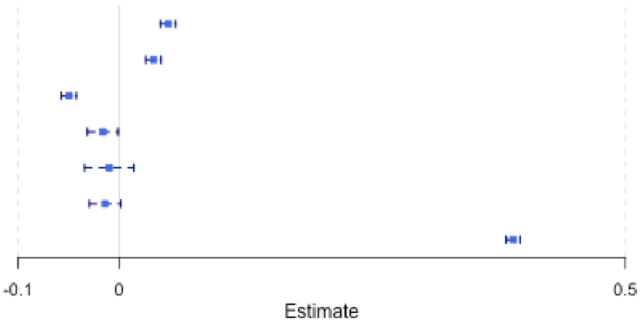

eFigure 6. Conventional epidemiological associations between potential confounders and birthweight.
Individuals included in this analysis are offspring with birthweight and at least one neurodevelopmental trait outcome available, as well as genotype data available for themselves and one or more parents. Estimates reflect standardised regression coefficients and 95% confidence intervals.

© 2023 D'Urso S et al. *JAMA Psychiatry*.

eAppendix 5. Proxy SNP identification

The LDmatrix tool (with the Utah Residents from North and West Europe (CEU) reference population) was used to identify proxy SNPs for missing variants[50]. Three SNPs in high linkage disequilibrium (LD) ($r^2 > 0.8$) were selected as proxies for missing variants (eTable 2). We were unable to identify proxies for some of the variants missing in the MoBa cohort after QC. We also used the LDmatrix tool and the same reference population to explore the LD between all variants used in the MR analyses and confirmed that the SNPs were independently associated with birthweight (all pairwise $r^2 < 0.01$).

eTable 2. Genetic variants used to proxy missing birthweight variants in MoBa.
Proxy variants were identified using the LDmatrix tool (r2 > 0.8) with Utah Residents from North and West Europe (CEU) reference population.

Original SNP	Proxy SNP	Correlated Alleles	Present in which allele scores?
rs181451002	rs56053206	G=T, A=C	A1, A2, A3, B2, B3
rs186606513	rs55908912	G=C, A=A	A1, B1, B3
rs255773	rs255774	C=A, T=G	A1, B1, B3

© 2023 D'Urso S et al. *JAMA Psychiatry*.

eAppendix 6. Missing genotype handling

For individuals who were missing a birthweight SNP, the mean dosage (from the founders, i.e., parents) was imputed across the entire triad/dyad/. Mean dosage imputation of missing genotype data would have added uncertainty to the measure of SNP dosage, and can lead to regression dilution bias, shifting the effect estimate obtained in the MR analyses towards the null. However, we believe the extent of this bias to be small, since only 0.5% of allele dosages were missing across all individuals. In addition, the missingness was not limited to particular imputation or genotyping batches, meaning that the imputation was non-systematic. While it is possible to apply a more complex approach and impute SNPs at a one-by-one basis (i.e., using allele frequencies and information from relatives), this would be computationally intensive.

© 2023 D'Urso S et al. *JAMA Psychiatry*.

eAppendix 7. SNP and allele score benchmarking

Various benchmarking checks were performed to investigate the validity of the allele scores used in the MR analysis (i.e., MR relevancy assumption). Firstly, we attempted to replicate the association between each SNP included in the allele scores and both own birthweight and offspring birthweight in MoBa, using linear regression analyses while adjusting for gestational duration, sex, birth year, genotyping batch and the first 10 genetic principal components (PCs) that were generated during the MoBaPsychGen QC process[36]. Most SNPs significant in the SEM analyses replicated in the conditional analysis ($p < 0.05$) (eTable 6).

Next, genetic linear mixed model analyses were conducted, to examine the association between birthweight allele scores and birthweight, whilst adjusting for offspring sex, offspring birth year, maternal and paternal age at birth, gestational duration and genotyping batches. A GRM, excluding birthweight SNPs and surroundings, was also included in the model. We demonstrate that offspring and maternal allele scores are associated with birthweight in the expected patterns (eTable 3). For example, maternal allele scores conditional on offspring/paternal allele scores (M1, M2, M3) are all significantly and positively associated with birthweight, while offspring allele scores conditional on maternal/paternal allele scores (F1, F2, F3) are also significantly and positively associated with birthweight. Paternal allele scores were not significantly associated with birthweight after conditioning on maternal and offspring allele scores (except for M1). In addition, for all the scores reflecting maternal genetic effects (M1, M2, M3), the maternal allele scores are more strongly associated with birthweight than offspring and paternal scores, whereas for the allele scores reflecting offspring genetic effects (F1, F2, F3), offspring scores are more strongly associated with birthweight than maternal allele scores.

Instrument strength was assessed using a conditional F-statistic, calculated from the same genetic linear mixed models described above (eTable 3). Two-sided P-values from the conditional effect of each allele score on offspring birthweight were converted to F-statistics involving 1 and $N – k – 1$ degrees of freedom, where k is the number of fixed-effects estimated in the model. For all allele scores of interest, the F-statistics were much larger than the recommended threshold of 10.

The variance in birthweight explained by the allele scores conditional on other covariates in the model (eTable 3) suggests that the offspring scores (F1, F2, F3) are better powered than the maternal scores (M1, M2, M3) to detect association, in line with past investigations into variance explained by maternal and fetal birthweight variants (see Discussion)[27].

© 2023 D’Urso S et al. *JAMA Psychiatry*.

eTable 3. Association between offspring, maternal and paternal birthweight alleles scores and Z-transformed birthweight. Genetic linear mixed models were used to examine the association between birthweight allele scores and birthweight, whilst adjusting for offspring sex, offspring birth year, maternal and paternal age at birth, gestational duration and genotyping batches. A GRM was also included in the model. CI = 95% confidence intervals. Bolded p-values < 0.05. Var(BW) is the variance explained in birthweight by the allele scores conditional on other covariates in the model. * denotes F-statistics and Var(BW) of interest

Allele Score	Predictor	Beta	Lower CI	Upper CI	P-Value	F-Statistic	Var(BW)
M1) SNPs associated with own or offspring birthweight	Offspring	0.003	0.002	0.004	**3.98E-06**	21.3	7.19E-04
	Maternal	0.006	0.005	0.007	**1.27E-28**	123.4*	3.22E-03*
	Paternal	0.001	0.000	0.002	**2.64E-02**	4.9	1.28E-04
M2) SNPs associated with offspring birthweight	Offspring	0.007	0.005	0.009	**6.09E-09**	33.8	1.14E-03
	Maternal	0.013	0.011	0.015	**2.54E-36**	158.7*	4.05E-03*
	Paternal	0.001	-0.001	0.003	1.67E-01	1.9	4.96E-05
M3) SNPs with a maternal-only effect on offspring birthweight	Offspring	0.007	0.003	0.010	**3.10E-04**	13.0	4.55E-04
	Maternal	0.018	0.014	0.021	**5.00E-28**	120.7*	3.19E-03*
	Paternal	0.002	-0.001	0.005	2.04E-01	1.6	4.24E-05
F1) SNPs associated with own or offspring birthweight	Offspring	0.013	0.011	0.014	**1.22E-84**	381.9*	1.28E-02*
	Maternal	-0.001	-0.002	0.000	2.39E-01	1.4	3.54E-05
	Paternal	0.000	-0.001	0.001	5.79E-01	0.3	7.88E-06
F2) SNPs associated with own birthweight	Offspring	0.018	0.017	0.020	**1.48E-104**	474.4*	1.58E-02*
	Maternal	0.000	-0.001	0.002	7.24E-01	0.1	3.17E-06
	Paternal	0.001	-0.001	0.002	2.73E-01	1.2	3.08E-05
F3) SNPs with fetal-only effects on offspring birthweight	Offspring	0.020	0.018	0.022	**1.34E-58**	261.4*	8.52E-03*
	Maternal	-0.002	-0.004	0.001	1.37E-01	2.2	5.53E-05
	Paternal	0.000	-0.002	0.002	8.21E-01	0.1	1.28E-06

© 2023 D'Urso S et al. *JAMA Psychiatry*.

eAppendix 8. Principal Component Analysis

Due to the high correlation between the offspring neurodevelopmental outcomes, Bonferroni correction for all 20 outcomes would be overly conservative. Instead, we performed a PCA in 15,719 offspring with measures available for all 20 neurodevelopmental traits to determine the number of PCs that explained more than 80% of the covariance between the offspring neurodevelopmental traits[55]. We determined that 10 PCs accounted for 80% of the covariance and applied a Bonferroni correction based on this number instead. This resulted in a multiple-testing corrected p-value threshold of $p < 0.005$ for statistical significance (eTable 4). Analyses were performed in R using the prcomp function.

eTable 4. Principal components analysis to determine the number of independent traits assessed in the present study.

**Principal Component**	**Eigenvalue**	**Proportion of Variance Explained**	**Cumulative Proportion of Variance Explained**
PC1	5.35	0.27	0.27
PC2	2.33	0.12	0.38
PC3	1.78	0.09	0.47
PC4	1.61	0.08	0.55
PC5	1.15	0.06	0.61
PC6	1.02	0.05	0.66
PC7	0.88	0.04	0.71
PC8	0.86	0.04	0.75
PC9	0.77	0.04	0.79
PC10	0.68	0.03	0.82
PC11	0.65	0.03	0.85
PC12	0.63	0.03	0.89
PC13	0.58	0.03	0.91
PC14	0.52	0.03	0.94
PC15	0.49	0.02	0.97
PC16	0.39	0.02	0.98
PC17	0.30	0.01	1.00
PC18	0.01	0.00	1.00
PC19	0.01	0.00	1.00
PC20	0.00	0.00	1.00

© 2023 D'Urso S et al. *JAMA Psychiatry*.

eAppendix 9. Power Calculations

We investigated the power to detect genetic effects in a MR study consisting of complete parent-offspring trios and maternal-dyads, under various scenarios, using the Power Calculator for Parent Offspring Trios With Complete Genotype Information[53] and the Maternal and Offspring Genetic Effects Power Calculator[54].

The relationship between maternal genetic effect, which is the proportion of variance in the trait explained by maternal genetic effects at the loci, offspring genetic effects, sample size and statistical power is presented in eFigure 7, eFigure 8 and eFigure 9. The power calculations found that the mother-offspring dyad analyses (N = 40,000) were better powered than trio analyses (N = 30,000) to detect both maternal genetic effects and offspring genetic effects (eFigure 7 and eFigure 8). However, trio analyses were still conducted to enable unbiased comparison between maternal genetic effect and paternal genetic effect estimates to understand the role of the postnatal environment on offspring NDDs. Furthermore, power to detect a maternal (or offspring genetic effect) does not differ in the presence of an offspring genetic effect (or maternal genetic effect). Under a multiple-testing correction for 10 tests (i.e., 10 independent offspring NDDs in the PCA), we had 80% power to detect a maternal (or fetal) genetic effect of 0.044% in 40,000 mother-offspring dyads (eFigure 9).

The power calculations suggest that we have ≥ 80% power to detect a maternal genetic effect that explained as little as 0.026% of the variance in offspring outcome (N = 40,000 dyads; offspring genetic effect = 0; two tailed alpha = 0.05; eFigure 7). For the trio analyses, we had ≥ 80% power to detect a maternal genetic effect that explained 0.039% of the variance in offspring outcome (N = 30,000 trios; offspring genetic effect = 0; two tailed alpha = 0.05; eFigure 7). We had 80% power to detect an offspring genetic effect that explained 0.026% and 0.052% of the variance in offspring outcome for maternal-dyad and trio analyses respectively (N = 40,000 dyads or 30,000 trios; maternal genetic effect = 0; two tailed alpha = 0.05; eFigure 8).

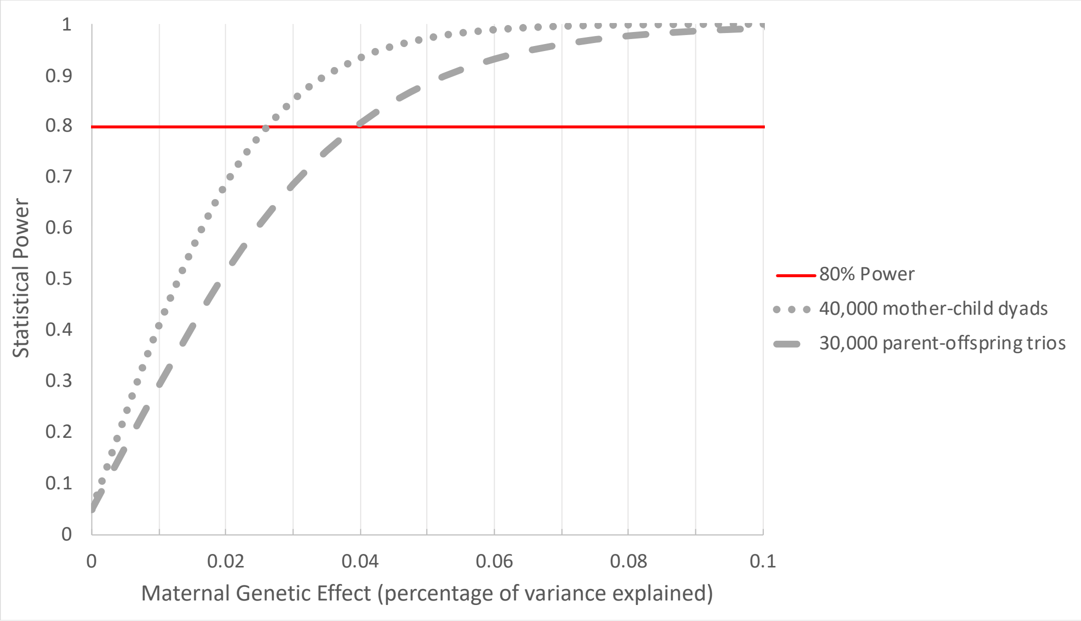

eFigure 7. Maternal genetic effect power calculations for mother-child dyad (N = 40,000) and parent-offspring trio (N = 30,000) analyses.

© 2023 D'Urso S et al. *JAMA Psychiatry*.

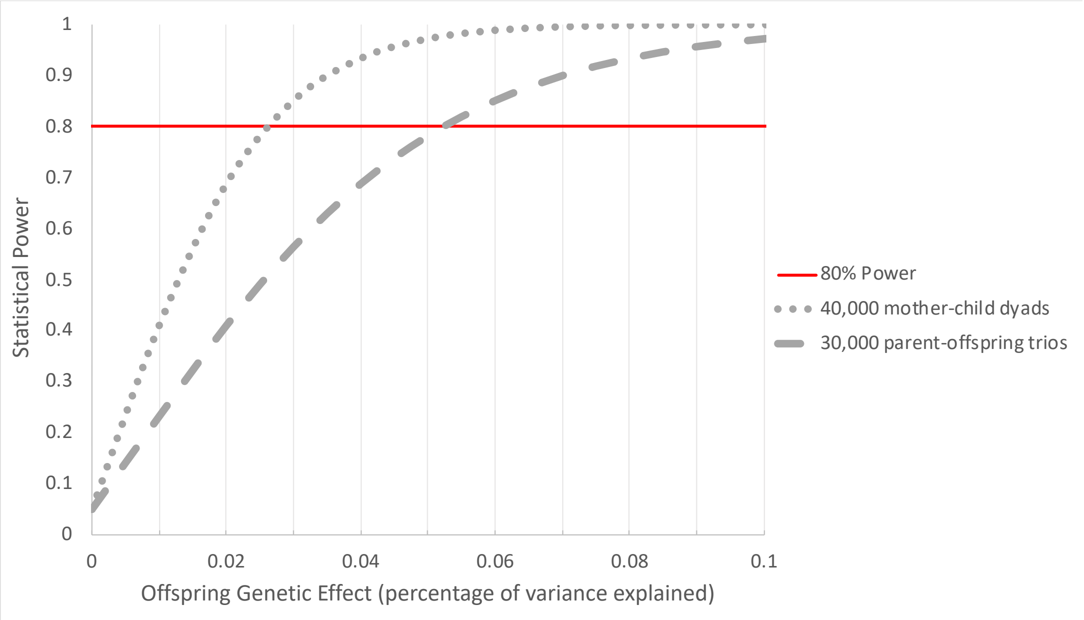

eFigure 8. Offspring genetic effect power calculations for mother-child dyad (N = 40,000) and parent-offspring trio (N = 30,000) analyses.

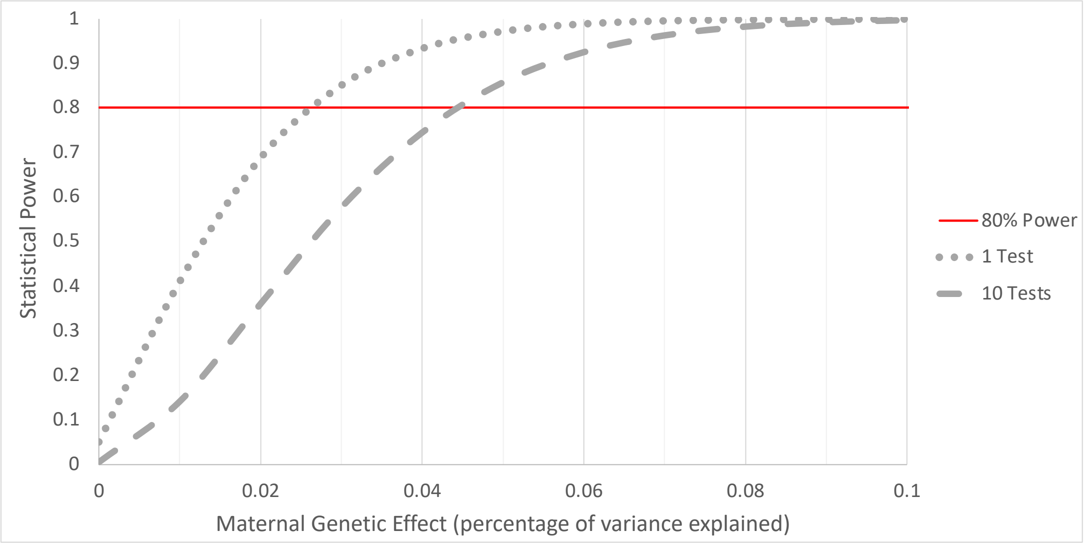

eFigure 9. Maternal genetic effect power calculations for mother-child dyad analyses ((N = 40,000) demonstrating the power of the study when performing one statistical test compared to 10 (alpha = 0.05 and 0.005).
The power for detecting a fetal genetic effect (rather than maternal) is identical.

© 2023 D'Urso S et al. *JAMA Psychiatry*.

eTable 5. Phenotypic and genetic characteristics of the genotyped MoBa offspring, mothers and fathers after QC.

	**Parent-Offspring Trios**			**Mother-Child Dyads**			**Father-Child Dyads**		
**Characteristic**	**Mean (SD)**	**Range**	**N***	**Mean (SD)**	**Range**	**N***	**Mean (SD)**	**Range**	**N***
Offspring Phenotypic Characteristics									
Year of birth (median)	2005	1999-2009	39,244	2005	1999-2009	60,856	2005	1999-2009	43,265
Sex (% male)	50.9		39,244	51		60,856	50.9		43,265
Birthweight (grams)	3673 (453)	2501-4990	39,231	3676 (456)	2501-4990	60,835	3674 (453)	2501-4990	43,252
Gestational age (weeks)	40 (1.3)	37-47	39,130	40 (1.3)	37-47	60,668	40 (1.3)	37-47	43,137
SCQ-full (3yr)	6.1 (3.3)	0-31	22,985	6.1 (3.3)	0-31	34,163	6.1 (3.3)	0-31	25,339
SCQ-full (8yr)	3.3 (2.8)	0-36	17,170	3.3 (2.9)	0-36	25,060	3.3 (2.8)	0-36	18,941
SCQ-RRB (3yr)	3.8 (2.5)	0-12	22,942	3.8 (2.5)	0-12	34,097	3.7 (2.5)	0-12	25,293
SCQ-RRB (8yr)	0.6 (1.1)	0-12	17,258	0.6 (1.1)	0-12	25,183	0.6 (1.1)	0-12	19,031
SCQ-SCI (3yr)	2.2 (1.7)	0-22	22,984	2.3 (1.8)	0-23	34,162	2.2 (1.7)	0-23	25,338
SCQ-SCI (8yr)	2.6 (2.4)	0-25	17,159	2.6 (2.4)	0-25	25,045	2.5 (2.4)	0-25	18,929
CBCL-ADHD (18m)	2.6 (1.6)	0-8	27,873	2.6 (1.6)	0-8	41,125	2.6 (1.6)	0-8	30,732
CBCL-ADHD (3yr)	3.4 (2.2)	0-12	22,941	3.4 (2.2)	0-12	34,097	3.4 (2.2)	0-12	25,289
CBCL-ADHD (5yr)	2.5 (2.1)	0-12	16,784	2.5 (2.1)	0-12	23,939	2.5 (2.1)	0-12	18,444
RS-DBD-ADHD (8yr)	8.3 (7)	0-54	17,249	8.4 (7.1)	0-54	25,168	8.3 (7.0)	0-54	19,022
RS-DBD-INA (8yr)	4.9 (4)	0-27	17,250	4.9 (4.1)	0-27	25,168	4.9 (4.0)	0-27	19,023
RS-DBD-HYP (8yr)	3.5 (3.8)	0-27	17,243	3.5 (3.9)	0-27	25,159	3.4 (3.8)	0-27	19,016
CPRS-ADHD 5yr	4.3 (4.5)	0-36	16,788	4.3 (4.5)	0-36	23,940	4.2 (4.4)	0-36	18,450
ASQ-LANG (18m)	1.2 (1.5)	0-6	28,833	1.2 (1.5)	0-6	44,221	1.2 (1.5)	0-6	31,793
ASQ-LANG (3yr)	0.6 (1.1)	0-12	23,001	0.6 (1.1)	0-12	34,196	0.6 (1.1)	0-12	25,362
ASQ-LANG (5yr)	0.7 (1.2)	0-14	16,733	0.7 (1.2)	0-14	23,876	0.7 (1.2)	0-14	18,390
CCC-S (8yr)	4.5 (4.2)	0-37	17,208	4.6 (4.2)	0-37	25,091	4.5 (4.2)	0-37	18,980
ASQ-MOTOR (18m)	0.7 (1.3)	0-12	28,871	0.7 (1.2)	0-12	44,276	0.7 (1.3)	0-12	31,837
ASQ-MOTOR (3yr)	1.2 (1.3)	0-8	22,924	1.1 (1.3)	0-8	34,078	1.2 (1.3)	0-8	25,277
CDI-MOTOR (5yr)	0.8 (1.4)	0-12	16,776	0.8 (1.4)	0-12	23,927	0.8 (1.4)	0-12	18,436
Maternal Phenotypic Characteristics									
Age at birth (years)	30.1 (4.4)	16-46	39,244	30.1 (4.5)	16-46	60,855	30.1 (4.4)	16-46	43,262
Gross yearly income (NOK)**	266,742 (119,451)	0-500,000	36,263	259,472 (120,622)	0-500,000	55,749	266,186 (119,474)	0-500,000	39,989
Education (%)***	i)1.9<br>ii)16.3<br>iii)13.9<br>iv)43.0<br>v)24.9		35,535	i)2.3<br>ii)17.7<br>iii)14.8<br>iv)41.9<br>v)23.3		54,769	i)1.9<br>ii)16.3<br>iii)13.9<br>iv)43.1<br>v)24.8		39,205
Paternal Phenotypic Characteristics									
Age at birth (years)	32.5 (5.1)	18-61	39,234	32.6 (5.3)	18-61	60,735	32.5 (5.1)	18-61	43,255
Gross yearly income (NOK)**	351,084 (115,777)	0-500,000	35,309	344,983 (117,373)	0-500,000	53,873	350,866 (115,780)	0-500,000	38,925
Education (%)***	i)3.6<br>ii)29.5<br>iii)12.0<br>iv)29.4		34,316	i)4.6<br>ii)31.7<br>iii)12.2<br>iv)27.8		52,734	i)3.6<br>ii)29.6<br>iii)12.0<br>iv)29.4		37,838

© 2023 D'Urso S et al. *JAMA Psychiatry*.

	v)25.5			v)23.6			v)25.5		
Offspring Genetic Characteristics									
Unweighted M1	204.4 (8.9)	167.9-246.2	39244	204.3 (9)	167.9-246.2	60856	204.3 (9)	167.9-246.2	43265
Unweighted M2	69.3 (4.9)	47-90.8	39244	69.2 (4.9)	47-90.8	60856	69.3 (4.9)	47-90.8	43265
Unweighted M3	33.1 (3.2)	21-46	39244	33.1 (3.2)	21-46	60856	33.1 (3.2)	21-46	43265
Unweighted F1	193.1 (9)	158-238.5	39244	193.1 (9)	158-238.5	60856	193.1 (9)	158-238.5	43265
Unweighted F2	133.5 (6.9)	103.4-165.4	39244	133.5 (6.9)	103.4-165.4	60856	133.5 (6.9)	103.4-165.4	43265
Unweighted F3	51.3 (4.6)	33.7-70	39244	51.3 (4.6)	32.8-70	60856	51.3 (4.6)	33.7-70	43265
Weighted M1	3.1 (0.2)	2.4-3.8	39244	3.1 (0.2)	2.4-3.8	60856	3.1 (0.2)	2.4-3.8	43265
Weighted M2	2 (0.1)	1.4-2.5	39244	2 (0.1)	1.4-2.5	60856	2 (0.1)	1.4-2.5	43265
Weighted M3	1.2 (0.1)	0.8-1.7	39244	1.2 (0.1)	0.8-1.7	60856	1.2 (0.1)	0.8-1.7	43265
Weighted F1	3.4 (0.2)	2.7-4.2	39244	3.4 (0.2)	2.7-4.2	60856	3.4 (0.2)	2.7-4.2	43265
Weighted F2	2.9 (0.2)	2.3-3.6	39244	2.9 (0.2)	2.3-3.6	60856	2.9 (0.2)	2.3-3.6	43265
Weighted F3	1.3 (0.1)	0.8-1.8	39244	1.3 (0.1)	0.8-1.8	60856	1.3 (0.1)	0.8-1.8	43265
Maternal Genetic Characteristics									
Unweighted M1	204.3 (9)	168.6-239.4	39244	204.2 (9)	168.6-241.7	60856	204.3 (9)	168.6-239.4	39244
Unweighted M2	69.2 (4.9)	50.8-89.7	39244	69.2 (4.9)	50.8-89.7	60856	69.2 (4.9)	50.8-89.7	39244
Unweighted M3	33.1 (3.2)	20-45	39244	33.1 (3.2)	20-45	60856	33.1 (3.2)	20-45	39244
Unweighted F1	193.1 (9)	149.5-232	39244	193.1 (9)	149.5-232	60856	193.1 (9)	149.5-232	39244
Unweighted F2	133.4 (6.9)	104.3-161.8	39244	133.5 (6.9)	104.3-163.8	60856	133.4 (6.9)	104.3-161.8	39244
Unweighted F3	51.3 (4.6)	32-69.3	39244	51.3 (4.7)	31-72	60856	51.3 (4.6)	32-69.3	39244
Weighted M1	3.1 (0.2)	2.5-3.7	39244	3.1 (0.2)	2.5-3.7	60856	3.1 (0.2)	2.5-3.7	39244
Weighted M2	2 (0.1)	1.5-2.5	39244	2 (0.1)	1.5-2.5	60856	2 (0.1)	1.5-2.5	39244
Weighted M3	1.2 (0.1)	0.8-1.6	39244	1.2 (0.1)	0.8-1.6	60856	1.2 (0.1)	0.8-1.6	39244
Weighted F1	3.4 (0.2)	2.7-4.2	39244	3.4 (0.2)	2.7-4.2	60856	3.4 (0.2)	2.7-4.2	39244
Weighted F2	2.9 (0.2)	2.3-3.5	39244	2.9 (0.2)	2.2-3.5	60856	2.9 (0.2)	2.3-3.5	39244
Weighted F3	1.3 (0.1)	0.8-1.8	39244	1.3 (0.1)	0.8-1.8	60856	1.3 (0.1)	0.8-1.8	39244
Paternal Genetic Characteristics									
Unweighted M1	204.3 (8.9)	169-241.3	39244	204.3 (8.9)	169-241.3	39244	204.3 (8.9)	169-241.3	43265
Unweighted M2	69.3 (4.9)	47-88	39244	69.3 (4.9)	47-88	39244	69.3 (4.9)	47-88	43265
Unweighted M3	33.1 (3.2)	21-45.5	39244	33.1 (3.2)	21-45.5	39244	33.1 (3.2)	20-45.5	43265
Unweighted F1	193.1 (9)	155.2-230.5	39244	193.1 (9)	155.2-230.5	39244	193.1 (9)	155.2-230.5	43265
Unweighted F2	133.5 (6.9)	107-161.9	39244	133.5 (6.9)	107-161.9	39244	133.5 (6.9)	107-161.9	43265
Unweighted F3	51.4 (4.6)	32-70	39244	51.4 (4.6)	32-70	39244	51.4 (4.6)	32-70	43265
Weighted M1	3.1 (0.2)	2.5-3.8	39244	3.1 (0.2)	2.5-3.8	39244	3.1 (0.2)	2.5-3.8	43265
Weighted M2	2 (0.1)	1.4-2.5	39244	2 (0.1)	1.4-2.5	39244	2 (0.1)	1.4-2.5	43265
Weighted M3	1.2 (0.1)	0.8-1.6	39244	1.2 (0.1)	0.8-1.6	39244	1.2 (0.1)	0.8-1.6	43265
Weighted F1	3.4 (0.2)	2.7-4.2	39244	3.4 (0.2)	2.7-4.2	39244	3.4 (0.2)	2.7-4.2	43265
Weighted F2	2.9 (0.2)	2.3-3.6	39244	2.9 (0.2)	2.3-3.6	39244	2.9 (0.2)	2.3-3.6	43265
Weighted F3	1.3 (0.1)	0.8-1.8	39244	1.3 (0.1)	0.8-1.8	39244	1.3 (0.1)	0.8-1.8	43265

N: Sample size; SD: Standard deviation. *Trio, maternal dyad and paternal dyad groups overlap. NOK = Norwegian Krone. **Gross yearly income calculated as midpoint of income brackets (no income, <150,000, 150,000-199,999, 200,000-299,999, 300,000- 399,999, 400,000-499,999, > 500,000) as reported by mothers at the 15th week of gestation. ***Education reflects the highest level of education completed as reported by mothers at the 15th week of gestation (i) 9-year secondary school, ii) 1-2 years high school or vocational high school, iii) 3-year high school general studies or junior college, iv) regional technical college or 4-year university degree, v) more than 4 years (university or technical college)).

© 2023 D'Urso S et al. *JAMA Psychiatry*.

eAppendix 10. Investigation into potential paternal selection bias

An additional post-hoc analysis into potential paternal selection bias into MoBa was conducted to help explain the positive findings in the negative control parental exposure analyses. We hypothesise that if allele scores for birthweight are pleiotropically associated with neurodevelopmental traits, and if fathers' participation in MoBa is influenced by these same neurodevelopmental traits, then selection may act as a collider and induce a positive association between paternal allele scores for birthweight and offspring neurodevelopmental outcomes (eFigure 10).

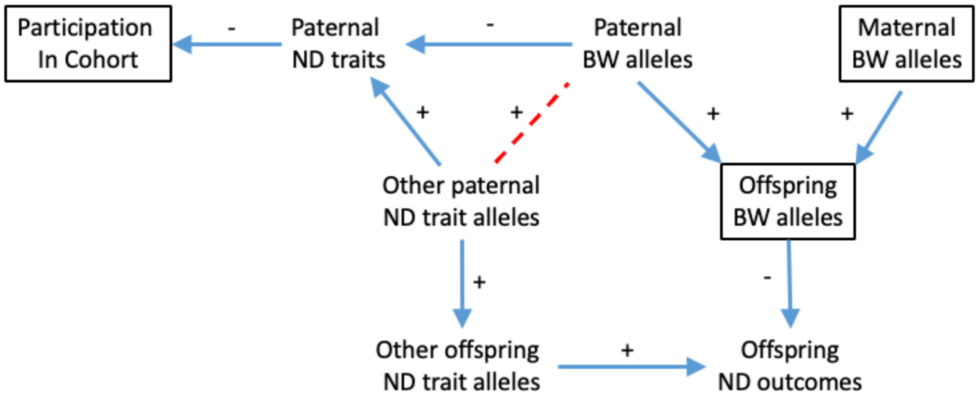

eFigure 10. Diagram illustrating the effect of selection bias on the parent-offspring trio study design for investigating the effect of an intrauterine exposure (proxied by birthweight; BW) on offspring neurodevelopmental (ND) outcomes.
If there is a relationship between paternal ND traits and participation in the cohort, a collider path (red dotted line) may open up and induce an association between paternal BW allele scores and offspring ND outcomes, even when conditioning on both offspring and maternal BW allele scores. Conditioned upon variables are outlined, whereas positive and negative associations are represented by + and – symbols, respectively.

We used adjusted and unadjusted linear regression analyses to assess whether father's presence in MoBa (i.e., the father was genotyped, and offspring was part of a parent-child trio or father-child dyad) was associated with the offspring NDDs. The adjusted model included offspring sex, health region of mother's place of residence, and maternal-age as covariates. The linear regression analyses found that paternal presence was associated with lower offspring neurodevelopmental outcome scores (eTable 6 adjusted analyses; $p < 0.005$; all SCQ scales, subscales and timepoints, CBCL-ADHD-18m, CBCL-ADHD-3yr, RSDBD-ADHD-8yr, ASQ-LANG-3yr and 5yr, and CCC-S-8yr). These analyses showed evidence to support this hypothesis; i.e., father's presence in MoBa (i.e., father is genotyped and offspring is part of a parent-offspring trio or father-offspring dyad) was associated with lower offspring neurodevelopmental outcomes in the expected direction (across all domains except for motor difficulties).

© 2023 D'Urso S et al. *JAMA Psychiatry*.

eTable 6. The relationship between paternal presence in MoBa and offspring neurodevelopmental trait outcomes. Linear regression analyses were used to test for an association between father's presence and the neurodevelopmental outcomes. The adjusted model included offspring sex, health region of mother's place of residence, and maternal age as covariates. Offspring who were part of a parent-offspring trio, mother-child dyad and father-child dyad were included in these analyses. Bolded p-values < 0.005.

		Unadjusted				Adjusted			
**Domain**	**Neurodevelopmental Outcome**	**Estimate**	**SE**	**P**	**N**	**Estimate**	**SE**	**P**	**N**
Difficulties with social communication and behavioural flexibility (repetitive behaviours)	SCQ-full (3yr)	-0.107	0.018	**3.46E-09**	38243	-0.128	0.018	**3.34E-13**	40043
	SCQ-full (8yr)	-0.127	0.022	**1.60E-08**	28145	-0.122	0.022	**2.24E-08**	29453
	SCQ-RRB (3yr)	-0.063	0.018	**5.05E-04**	38173	-0.087	0.018	**8.41E-07**	39971
	SCQ-RRB (8yr)	-0.084	0.022	**1.81E-04**	28279	-0.084	0.022	**1.32E-04**	29594
	SCQ-SCI (3yr)	-0.105	0.018	**7.73E-09**	38241	-0.108	0.018	**9.70E-10**	40041
	SCQ-SCI (8yr)	-0.103	0.022	**4.21E-06**	28129	-0.097	0.022	**1.01E-05**	29437
Difficulties with attention and hyperactive-impulsive behaviour	CBCL-ADHD (18m)	-0.061	0.017	**2.99E-04**	46020	-0.066	0.016	**5.27E-05**	48174
	CBCL-ADHD (3yr)	-0.036	0.018	4.49E-02	38164	-0.051	0.018	**4.40E-03**	39960
	CBCL-ADHD (5yr)	-0.021	0.024	0.383	26810	-0.028	0.023	2.37E-01	28027
	RS-DBD-ADHD (8yr)	-0.057	0.022	1.07E-02	28262	-0.061	0.022	**4.81E-03**	29575
	RS-DBD-INA (8yr)	-0.044	0.022	4.95E-02	28262	-0.053	0.022	1.37E-02	29574
	RS-DBD-HYP (8yr)	-0.059	0.022	8.78E-02	28253	-0.056	0.022	1.00E-02	29566
	CPRS (5yr)	-0.023	0.024	0.329	26811	-0.039	0.023	9.27E-02	28032
Difficulties with language	ASQ-LANG (18m)	-0.028	0.015	0.058	49492	-0.025	0.014	7.52E-02	51803
	ASQ-LANG (3yr)	-0.121	0.018	**3.01E-11**	38282	-0.114	0.018	**1.03E-10**	40078
	ASQ-LANG (5yr)	-0.068	0.024	**4.71E-03**	26740	-0.068	0.023	**3.56E-03**	27952
	CCC-S (8yr)	-0.120	0.022	**1.04E-07**	28182	-0.115	0.022	**1.71E-07**	29490
Difficulties with motor skills	ASQ-MOTOR (18m)	-0.031	0.015	3.44E-02	49554	-0.028	0.014	5.25E-02	51867
	ASQ-MOTOR (3yr)	0.011	0.018	0.543	38150	-0.002	0.017	9.00E-01	39949
	CDI-MOTOR (5yr)	-0.043	0.024	0.072	26795	0.043	0.023	6.03E-02	28018

Abbreviations: SE = Standard Error

© 2023 D'Urso S et al. *JAMA Psychiatry*.

References

1. Forsdahl A. Are poor living conditions in childhood and adolescence an important risk factor for arteriosclerotic heart disease? *Br J Prev Soc Med*. 1977;31(2):91-95. doi:10.1136/jech.31.2.91
2. Barker DJ, Winter PD, Osmond C, Margetts B, Simmonds SJ. Weight in infancy and death from ischaemic heart disease. *Lancet*. 1989;2(8663):577-580. doi:10.1016/s0140-6736(89)90710-1
3. Barker DJ, Osmond C, Simmonds SJ, Wield GA. The relation of small head circumference and thinness at birth to death from cardiovascular disease in adult life. *BMJ : British Medical Journal*. 1993;306(6875):422-426. https://www.ncbi.nlm.nih.gov/pmc/articles/PMC1676496/
4. Barker DJ, Osmond C. Infant mortality, childhood nutrition, and ischaemic heart disease in England and Wales. *Lancet*. 1986;1(8489):1077-1081. doi:10.1016/s0140-6736(86)91340-1
5. Suzuki K. The developing world of DOHaD. *J Dev Orig Health Dis*. 2018;9(3):266-269. doi:DOI: 10.1017/S2040174417000691
6. O'Donnell KJ, Meaney MJ. Fetal Origins of Mental Health: The Developmental Origins of Health and Disease Hypothesis. *Am J Psychiatry*. 2017;174(4):319-328. doi:10.1176/appi.ajp.2016.16020138
7. Murray E, Fernandes M, Fazel M, Kennedy SH, Villar J, Stein A. Differential effect of intrauterine growth restriction on childhood neurodevelopment: a systematic review. *BJOG*. 2015;122(8):1062-1072. doi:https://doi.org/10.1111/1471-0528.13435
8. Levine TA, Grunau RE, McAuliffe FM, Pinnamaneni R, Foran A, Alderdice FA. Early Childhood Neurodevelopment After Intrauterine Growth Restriction: A Systematic Review. *Pediatrics*. 2015;135(1):126-141. doi:10.1542/peds.2014-1143
9. Levine TA, Grunau RE, McAuliffe FM, Alderdice FA. Early psychosocial development of small for gestational age and intrauterine growth-restricted children: a systematic review. *Journal of Perinatology*. 2019;39(8):1021-1030. doi:10.1038/s41372-019-0369-y
10. Kenny L, Hattersley C, Molins B, Buckley C, Povey C, Pellicano E. Which terms should be used to describe autism? Perspectives from the UK autism community. *Autism*. 2016;20(4):442-462.
11. Anderson PJ, de Luca CR, Hutchinson E, et al. Attention problems in a representative sample of extremely preterm/extremely low birth weight children. *Dev Neuropsychol*. 2011;36(1):57-73. doi:10.1080/87565641.2011.540538
12. Baron IS, Kerns KA, Müller U, Ahronovich MD, Litman FR. Executive functions in extremely low birth weight and late-preterm preschoolers: effects on working memory and response inhibition. *Child Neuropsychol*. 2012;18(6):586-599. doi:10.1080/09297049.2011.631906
13. Bhutta AT, Cleves MA, Casey PH, Cradock MM, Anand KJS. Cognitive and Behavioral Outcomes of School-Aged Children Who Were Born Preterm A Meta-analysis. *JAMA*. 2002;288(6):728-737. doi:10.1001/jama.288.6.728
14. Elgen I, Sommerfelt K, Markestad T. Population based, controlled study of behavioural problems and psychiatric disorders in low birthweight children at 11 years of age. *Arch Dis Child Fetal Neonatal Ed*. 2002;87(2):F128. doi:10.1136/fn.87.2.F128
15. Madigan S, Wade M, Plamondon A, Browne D, Jenkins JM. Birth Weight Variability and Language Development: Risk, Resilience, and Responsive Parenting. *J Pediatr Psychol*. 2015;40(9):869-877. doi:10.1093/jpepsy/jsv056
16. Ment LR, Vohr B, Allan W, et al. Change in cognitive function over time in very low-birth-weight infants. *JAMA*. 2003;289(6):705-711. doi:10.1001/jama.289.6.705
17. van Houdt CA, Oosterlaan J, van Wassenaer-Leemhuis AG, van Kaam AH, Aarnoudse-Moens CSH. Executive function deficits in children born preterm or at low birthweight: a meta-analysis. *Dev Med Child Neurol*. 2019;61(9):1015-1024. doi:10.1111/dmcn.14213
18. Momany AM, Kamradt JM, Nikolas MA. A Meta-Analysis of the Association Between Birth Weight and Attention Deficit Hyperactivity Disorder. *J Abnorm Child Psychol*. 2018;46(7):1409-1426. doi:10.1007/s10802-017-0371-9
19. Lampi KM, Lehtonen L, Tran PL, et al. Risk of Autism Spectrum Disorders in Low Birth Weight and Small for Gestational Age Infants. *J Pediatr*. 2012;161(5):830-836. doi:https://doi.org/10.1016/j.jpeds.2012.04.058
20. Sciberras E, Mulraney M, Silva D, Coghill D. Prenatal Risk Factors and the Etiology of ADHD—Review of Existing Evidence. *Curr Psychiatry Rep*. 2017;19(1):1. doi:10.1007/s11920-017-0753-2
21. Levine TA, Grunau RE, McAuliffe FM, Pinnamaneni R, Foran A, Alderdice FA. Early Childhood Neurodevelopment After Intrauterine Growth Restriction: A Systematic Review. *Pediatrics*. 2015;135(1):126-141. doi:10.1542/peds.2014-1143
22. Gage SH, Munafò MR, Davey Smith G. Causal Inference in Developmental Origins of Health and Disease (DOHaD) Research. *Annu Rev Psychol*. 2016;67(1):567-585. doi:10.1146/annurev-psych-122414-033352

© 2023 D'Urso S et al. *JAMA Psychiatry*.

23. Davey Smith G, Ebrahim S. 'Mendelian randomization': can genetic epidemiology contribute to understanding environmental determinants of disease?*. *Int J Epidemiol*. 2003;32(1):1-22. doi:10.1093/ije/dyg070
24. Davey Smith G, Lawlor DA, Harbord R, Timpson N, Day I, Ebrahim S. Clustered environments and randomized genes: a fundamental distinction between conventional and genetic epidemiology. *PLoS Med*. 2007;4(12):e352. doi:10.1371/journal.pmed.0040352
25. Tyrrell J, Richmond RC, Palmer TM, et al. Genetic Evidence for Causal Relationships Between Maternal Obesity-Related Traits and Birth Weight. *JAMA*. 2016;315(11):1129-1140. doi:10.1001/jama.2016.1975
26. Evans DM, Moen GH, Hwang LD, Lawlor DA, Warrington NM. Elucidating the role of maternal environmental exposures on offspring health and disease using two-sample Mendelian randomization. *Int J Epidemiol*. 2019;48(3):861-875. doi:10.1093/ije/dyz019
27. Warrington NM, Beaumont RN, Horikoshi M, et al. Maternal and fetal genetic effects on birth weight and their relevance to cardio-metabolic risk factors. *Nat Genet*. 2019;51(5):804-814. doi:10.1038/s41588-019-0403-1
28. Wang G, Bhatta L, Moen GH, et al. Investigating a Potential Causal Relationship Between Maternal Blood Pressure During Pregnancy and Future Offspring Cardiometabolic Health. *Hypertension*. 2022;79(1):170-177. doi:10.1161/HYPERTENSIONAHA.121.17701
29. Moen GH, Brumpton B, Willer C, et al. Mendelian randomization study of maternal influences on birthweight and future cardiometabolic risk in the HUNT cohort. *Nat Commun*. 2020;11(1):5404. doi:10.1038/s41467-020-19257-z
30. Caramaschi D, Taylor AE, Richmond RC, et al. Maternal smoking during pregnancy and autism: using causal inference methods in a birth cohort study. *Transl Psychiatry*. 2018;8(1):262. doi:10.1038/s41398-018-0313-5
31. Zerbo O, Traglia M, Yoshida C, et al. Maternal mid-pregnancy C-reactive protein and risk of autism spectrum disorders: the early markers for autism study. *Transl Psychiatry*. 2016;6(4):e783-e783. doi:10.1038/tp.2016.46
32. Lawlor D, Richmond R, Warrington N, et al. Using Mendelian randomization to determine causal effects of maternal pregnancy (intrauterine) exposures on offspring outcomes: Sources of bias and methods for assessing them. *Wellcome Open Res*. 2017;2:11. doi:10.12688/wellcomeopenres.10567.1
33. Magnus P, Birke C, Vejrup K, et al. Cohort Profile Update: The Norwegian Mother and Child Cohort Study (MoBa). *Int J Epidemiol*. 2016;45(2):382-388. doi:10.1093/ije/dyw029
34. Magnus P, Irgens LM, Haug K, et al. Cohort profile: The Norwegian Mother and Child Cohort Study (MoBa). *Int J Epidemiol*. 2006;35(5):1146-1150. doi:10.1093/ije/dyl170
35. Paltiel L, Anita H, Skjerden T, et al. The biobank of the Norwegian Mother and Child Cohort Study–present status. *Norsk epidemiologi*. 2014;24(1-2).
36. Corfield EC, Frei O, Shadrin AA, et al. The Norwegian Mother, Father, and Child cohort study (MoBa) genotyping data resource: MoBaPsychGen pipeline v.1. *bioRxiv*. Published online January 1, 2022:2022.06.23.496289. doi:10.1101/2022.06.23.496289
37. Rutter M, Bailey A, Lord C. The social communication questionnaire: manual. *Torrance, CA: WPS*. Published online 2003.
38. Achenbach TM. *Manual for the Child Behavior Checklist/2-3 and 1992 Profile*. Dept. of Psychiatry, University of Vermont; 1992.
39. Silva RR, Alpert M, Pouget E, et al. A Rating Scale for Disruptive Behavior Disorders, Based on the DSM-IV Item Pool. *Psychiatric Quarterly*. 2005;76(4):327-339. doi:10.1007/s11126-005-4966-x
40. Conners CK, Sitarenios G, Parker JDA, Epstein JN. The Revised Conners' Parent Rating Scale (CPRS-R): Factor Structure, Reliability, and Criterion Validity. *J Abnorm Child Psychol*. 1998;26(4):257-268. doi:10.1023/A:1022602400621
41. Janson H, Smith L. Norsk manual supplement til Ages and Stages Questionnaires. *Oslo: Regionsenter for barne- og ungdomspsykiatri, Helseregion Øst/Sør*. Published online 2003.
42. Richter J, Janson H. A validation study of the Norwegian version of the Ages and Stages Questionnaires. *Acta Paediatr*. 2007;96(5):748-752. Accessed December 8, 2022. https://www.academia.edu/25490194/A_validation_study_of_the_Norwegian_version_of_the_Ages_and_Stages_Questionnaires
43. Squires J, Bricker DD, Potter L. *The ASQ User's Guide*. 2nd ed. Paul H. Brookes; 1999.
44. Norbury CF, Nash M, Baird G, Bishop DVM. Using a parental checklist to identify diagnostic groups in children with communication impairment: a validation of the Children's Communication Checklist—2. *Int J Lang Commun Disord*. 2004;39(3):345-364.
45. Bishop DVM. *The Children's Communication Checklist*. Vol 2. Psychological Corporation London; 2003.
46. Ireton H. *The Child Development Inventory Manual*.; 1992.

© 2023 D'Urso S et al. *JAMA Psychiatry*.

47. Chaffee CA, Cunningham CE, Secord-Gilbert M, Elbard H, Richards J. Screening effectiveness of the Minnesota Child Development Inventory expressive and receptive language scales: Sensitivity, specificity, and predictive value. *Psychological Assessment: A Journal of Consulting and Clinical Psychology*. 1990;2(1):80.
48. Yang J, Lee SH, Goddard ME, Visscher PM. GCTA: A Tool for Genome-wide Complex Trait Analysis. *The American Journal of Human Genetics*. 2011;88(1):76-82. doi:https://doi.org/10.1016/j.ajhg.2010.11.011
49. Yang J, Benyamin B, McEvoy BP, et al. Common SNPs explain a large proportion of the heritability for human height. *Nat Genet*. 2010;42:565. https://doi.org/10.1038/ng.608
50. Machiela MJ, Chanock SJ. LDlink: a web-based application for exploring population-specific haplotype structure and linking correlated alleles of possible functional variants. *Bioinformatics*. 2015;31(21):3555-3557. doi:10.1093/bioinformatics/btv402
51. Warrington NM, Freathy RM, Neale MC, Evans DM. Using structural equation modelling to jointly estimate maternal and fetal effects on birthweight in the UK Biobank. *Int J Epidemiol*. 2018;47(4):1229-1241. doi:10.1093/ije/dyy015
52. Lewis SJ, Relton C, Zammit S, Smith GD. Approaches for strengthening causal inference regarding prenatal risk factors for childhood behavioural and psychiatric disorders. *Journal of Child Psychology and Psychiatry*. 2013;54(10):1095-1108. doi:10.1111/jcpp.12127
53. Tubbs JD, Hwang LD, Luong J, Evans DM, Sham PC. Modeling Parent-Specific Genetic Nurture in Families with Missing Parental Genotypes: Application to Birthweight and BMI. *Behav Genet*. 2021;51(3):289-300. doi:10.1007/s10519-020-10040-w
54. Moen GH, Hemani G, Warrington NM, Evans DM. Calculating Power to Detect Maternal and Offspring Genetic Effects in Genetic Association Studies. *Behav Genet*. 2019;49(3):327-339. doi:10.1007/s10519-018-9944-9
55. Havdahl A, Wootton RE, Leppert B, et al. Associations Between Pregnancy-Related Predisposing Factors for Offspring Neurodevelopmental Conditions and Parental Genetic Liability to Attention-Deficit/Hyperactivity Disorder, Autism, and Schizophrenia: The Norwegian Mother, Father and Child Cohort Study (MoBa). *JAMA Psychiatry*. 2022;79(8):799-810. doi:10.1001/jamapsychiatry.2022.1728
56. Latimer K, Wilson P, Kemp J, et al. Disruptive behaviour disorders: a systematic review of environmental antenatal and early years risk factors. *Child Care Health Dev*. 2012;38(5):611-628. doi:https://doi.org/10.1111/j.1365-2214.2012.01366.x
57. D'Urso S, Wang G, Hwang LD, Moen GH, Warrington NM, Evans DM. A cautionary note on using Mendelian randomization to examine the Barker hypothesis and Developmental Origins of Health and Disease (DOHaD). *J Dev Orig Health Dis*. 2021;12(5):688-693. doi:10.1017/S2040174420001105
58. Burgess S, Davies NM, Thompson SG. Bias due to participant overlap in two-sample Mendelian randomization. *Genet Epidemiol*. 2016;40(7):597-608. doi:https://doi.org/10.1002/gepi.21998
59. Smith GD, Lipsitch M, Tchetgen ET, Cohen T. Negative Control Exposures in Epidemiologic Studies. *Epidemiology*. 2012;23(2):350-352. http://www.jstor.org/stable/23214345
60. Burgess S, Thompson SG. *Mendelian Randomization: Methods for Using Genetic Variants in Causal Estimation*. CRC Press; 2015.
61. Hanson MA, Gluckman PD. Early Developmental Conditioning of Later Health and Disease: Physiology or Pathophysiology? *Physiol Rev*. 2014;94(4):1027-1076. doi:10.1152/physrev.00029.2013

© 2023 D'Urso S et al. *JAMA Psychiatry*.
