# Supplementary material for: Intrauterine Growth and Offspring Neurodevelopmental Traits: A Mendelian Randomization Analysis of the Norwegian Mother, Father and Child Cohort Study (MoBa)
Source: JAMA Psychiatry. 2023 Oct 25;81(2):144–56. doi: 10.1001/jamapsychiatry.2023.3872 (PMC10600722; doi:10.1001/jamapsychiatry.2023.3872)
Supplement: Supplement 3. — Data Sharing Statement [file jamapsychiatry-e233872-s003.pdf]

Data Sharing Statement

D’Urso. Intrauterine Growth and Offspring Neurodevelopmental Traits. *JAMA Psychiatry*.
Published October 25, 2023. doi:10.1001/jamapsychiatry.2023.3872

Data
Data available: No
